# Selective targeting BMP2 and 4 in SMAD4 negative esophageal adenocarcinoma inhibits tumor growth and aggressiveness in preclinical models

**DOI:** 10.1007/s13402-022-00689-2

**Published:** 2022-07-28

**Authors:** Shulin Li, Sanne J. M. Hoefnagel, Matthew Read, Sybren Meijer, Mark I. van Berge Henegouwen, Suzanne S. Gisbertz, Elena Bonora, David S. H. Liu, Wayne A. Phillips, Silvia Calpe, Ana C. P. Correia, Maria D. C. Sancho-Serra, Sandro Mattioli, Kausilia K. Krishnadath, Members of the Esophageal Adenocarcinoma Study Group Europe (EACSGE)

**Affiliations:** 1 Center for Experimental and Molecular Medicine, Amsterdam UMC, University of Amsterdam, Amsterdam, the Netherlands; 2 Department of Gastroenterology and Hepatology, Amsterdam UMC, University of Amsterdam, Amsterdam, the Netherlands; 3 Department of Surgery, University of Melbourne, St Vincent’s Hospital, Melbourne, Australia; 4 Peter MacCallum Cancer Centre, Melbourne, Australia; 5 Department of Pathology, Amsterdam UMC, Cancer Center Amsterdam, University of Amsterdam, Amsterdam, the Netherlands; 6 Department of Surgery, Amsterdam UMC, University of Amsterdam, Cancer Center Amsterdam, Amsterdam, the Netherlands; 7 Department of Medical and Surgical Sciences, University of Bologna, U.O. Genetica Medica, IRCCS Azienda Ospedaliero-Universitaria di Bologna, Bologna, Italy; 8 Upper Gatrointestinal Unit, Department of Surgery, Austin Health, Heidelberg, Victoria Australia; 9 Division of Cancer Research, Peter MacCallum Cancer Centre, Parkville, Victoria Australia; 10 Sir Peter MacCallum Department of Oncology, University of Melbourne, Parkville, Australia; 11 Division of Thoracic Surgery, Maria Cecilia Hospital, GVM Care & Research Group, Cotignola, 48022, Ravenna, Italy; 12 Present Address: Department of Gastroenterology and Hepatology, University Hospital Antwerp, Antwerp, Belgium; 13 Laboratory of Experimental Medicine and Pediatrics, University of Antwerp, Antwerp, Belgium

**Keywords:** esophageal adenocarcinoma, SMAD4, BMP2, BMP4, anti-BMP antibodies, VHHs

## Abstract

**Purpose:**

Abnormalities within the Sonic Hedgehog (SHH), Bone Morphogenetic Protein (BMP) and SMAD4 signalling pathways have been associated with the malignant behavior of esophageal adenocarcinoma (EAC). We recently developed two specific llama-derived antibodies (VHHs), C4C4 and C8C8, which target BMP4 and BMP2/4, respectively. Here we aimed to demonstrate the feasibility of the VHHs for the treatment of EAC and to elucidate its underlying mechanism.

**Methods:**

Gene Set Enrichment Analysis (GSEA) was performed on a TCGA dataset, while expression of SHH, BMP2/4 and SMAD4 was validated in a cohort of EAC patients. The effects of the VHHs were tested on the recently established SMAD4(-) ISO76A primary EAC cell line and its counterpart SMAD4(+) ISO76A. In a patient-derived xenograft (PDX) model, the VHHs were evaluated for their ability to selectively target tumor cells and for their effects on tumor growth and survival.

**Results:**

High expression of BMP2/4 was detected in all SMAD4 negative EACs. SHH upregulated BMP2/4 expression and induced p38 MAPK signaling in the SMAD4(-) ISO76A cells. Inhibition of BMP2/4 by VHHs decreased the aggressive and chemo-resistant phenotype of the SMAD4(-) ISO76A but not of the SMAD4(+) ISO76A cells. In the PDX model, in vivo imaging indicated that VHHs effectively targeted tumor cells. Both VHHs significantly inhibited tumor growth and acted synergistically with cisplatin. Furthermore, we found that C8C8 significantly improved survival of the mice.

**Conclusions:**

Our data indicate that increased BMP2/4 expression triggers aggressive non-canonical BMP signaling in SMAD4 negative EAC. Inhibiting BMP2/4 decreases malignant behavior and improves survival. Therefore, VHHs directed against BMP2/4 hold promise for the treatment of SMAD4 negative EAC.

**Supplementary Information:**

The online version contains supplementary material available at 10.1007/s13402-022-00689-2.

## Introduction

Esophageal adenocarcinoma (EAC) is a highly metastatic disease associated with poor clinical outcomes. Overall, its 5-year survival rate is only 20%. In Western countries, a significant and sustained rise in the incidence of EAC has been observed. The introduction of neoadjuvant treatment in the form of either chemotherapy or the combination of chemo- and radiotherapy improved the survival of potentially curable cases to around 40% [1, 2]. Combining conventional therapy with inhibition of growth factors such as EGFR and ERBB2 has slightly improved outcomes [3], but a major breakthrough in treatment has not been reached. A major problem is that none of the existing molecular therapies has been specifically developed for EAC. Therefore, novel and effective molecular targeting treatments specifically for EAC are an unmet clinical need.

The SMAD4 tumor suppressor gene is pivotal for downstream signaling of Bone Morphogenetic Proteins (BMPs). This pathway is activated by upstream ligands such as Sonic Hedgehog (SHH) [4, 5]. Importantly, SMAD4 is frequently lost in gastrointestinal cancer [6, 7] and in 8 to 10 % of EAC [8–10]. SMAD4 loss is associated with non-canonical BMP signaling leading to a more metastatic phenotype, a poor prognosis and a poor response to treatment [11]. Similar observations have been reported for aberrant activation of SHH, the upstream BMP ligand, in cancers of the pancreas, stomach and colon [12]. SHH produced by cancer-associated fibroblasts (CAFs) in the microenvironment has been demonstrated to regulate cancer progression to trigger metastasis and chemoresistance [12].

BMP2 and BMP4 (BMP2/4) are critically involved in malignant signaling leading to cancer progression [13] and are well-known for their involvement in metastatic and invasive behavior of cancer [14–18]. In hepatocellular carcinoma [19, 20] gastric [21, 22] and colon cancer [23, 24]. BMP2 contributes to tumor cell migration, invasiveness and metastasis. Upregulated BMP4 has been reported in gastric cancer, hepatocellular carcinoma and colorectal cancer [25–27]. Compared to normal esophageal squamous epithelium, BMP4 expression is also significantly upregulated in EAC and its precursor lesion, Barrett’s Esophagus (BE) [28, 29]. Recently, our group demonstrated in a murine model that inhibition of BMPs effectively inhibits the regeneration of columnar epithelium and promotes the development of a neo-squamous epithelium from stem cells residing at the squamo-columnar junction (SCJ) following the ablation of normal columnar epithelium in this region [30].

Focusing on BMP2/4, we recently developed two specific antibodies, C4C4 and C8C8, targeting BMP4 and BMP2/4, respectively. VHHs are Llama-derived single domain antibodies, which are low molecular weight molecules of around 15 kDa. As opposed to conventional antibodies, VHHs’ antigen-binding fragment is formed by only the variable domain of the heavy chain. This enables VHHs to bind specifically and with high affinity to their associated antigen as well as to hidden epitopes within grooves or cavities. Notably, the world’s first VHH applied in the clinic, caplacizumab, was approved in Europe and the US in 2018 for patients with acquired thrombotic thrombocytopenic purpura [31]. The US Food and Drug Administration (FDA) considers it to be a first-in-class medication [32]. Moreover, studies have shown that VHH could serve as a potential anti-COVID-19 agent because of its potent neutralizing ability and peculiar characteristics such as small size, low immunogenicity and high affinity and stability [33, 34]. Above all, our previous studies have demonstrated that our VHHs targeting BMP2/4 have high specificity and affinity and low off-target effects compared to conventional antibodies or antagonists [35, 36].

Although our previous studies have identified two superior antibodies targeted for BMP2/4, important questions remain to be studied. We hypothesized that SMAD4 loss is the driver for aggressive behavior in a subset of EAC and that it allows activation of malignant pathways when stimulated by BMP2/4. In this study, we set out to assess whether selective targeting of BMP2/4 could affect the process of malignant BMP signaling, cell migration, chemoresistance and growth in SMAD4 negative EAC. Importantly, we sought to test the feasibility of the VHHs for treatment of SMAD4 negative EAC. Toward these goals, BMP2/4 and SMAD4 pathways were interrogated using data from the TCGA as well as local patient samples. Confirmatory mechanistic studies were performed using a range of preclinical models.

## Materials and methods

### Ethics statement

This study was approved by the human ethics committee, the Peter MacCallum Cancer Centre Human Research Ethics Committee (08/30, 10/108 and/or 18/211). This study was approved by the animal ethics committee, the Peter MacCallum Cancer Centre Animal Experimentation Ethics Committee (E534 and E598).

### Data sources

Clinical and RNA expression data from patients with EAC from the TCGA cohort were retrieved using the R package TCGAbiolinks [37, 38]. Presence or absence of somatic mutations in the SMAD4 gene of tissue samples from the TCGA cohort were defined by results from the “muse” pipeline, and retrieved by the function GDCquery Maf [39]. Expression data of patients with and without SMAD4 mutations were visualized by the pheatmap [40]. This heatmap was restricted to show the expression of the 5000 most varying genes after normalization by variance stabilizing transformation by DESeq2 and calculation of z-scores [41].

First, a broad analysis was performed and the GSEA tool from the Broad Institute [42] was used to compare gene set enrichment for a large number of gene sets, derived from the REACTOME, KEGG and BIOCARTA databases, in patients with EAC from the TCGA cohort with and without a SMAD4 mutation [42–45]. Using 1000 permutations, significance of gene set enrichment was set to a nominal *p*-value < 0.05. Enrichment plots were shown for gene sets of interest, with a graphical view of the enrichment score per gene set. Thereafter, a subset of patients was selected for further analyses to investigate pathways associated with differences in SMAD4 mutation status and RNA expression. EAC patients with a SMAD4 mutation and a control cohort consisting of EAC patients which were sub-selected based on having the highest SMAD4 expression levels and without a SMAD4 mutation, were compared by differential expression analysis by DESeq2. Significance was set to an adjusted *p*-value of ≤ 0.05. A heatmap of these subsets of patients and the differentially expressed genes was visualized, with expression of these genes defined by applying z-scores on the raw count data. Results served as input for Qiagen’s Ingenuity Pathway Analysis. Significance was defined by a -log(*p*-value) of ≥ 1.3 and a zscore ≥ than 2.0 or ≤ than -2.0. Results from these analyses were visualized in the pheatmap. IPA pathways of interest were selected for visualization in a heatmap of their differentially expressed genes.

### Study population and human tissues

Archival formalin fixed paraffin embedded (FFPE) resection specimens of 40 EAC cases from the Amsterdam UMC treated by surgery between 2006 and 2011 were used for immunohistochemistry (IHC) to assess SHH, BMP2, BMP4 and SMAD4 expression and for targeted sequencing to detect SMAD4 mutations.

### Ethical considerations

For the use of the archival tissues from the Netherlands, the collection, storage and use of patient derived paraffin embedded tissue and data were performed in compliance with the “Code for Proper Secondary Use of Human Tissue in The Netherlands”, Dutch Federation of Biomedical Scientific Societies, the Netherlands and therefore no informed consent was required. For the fresh frozen biopsies from the Amsterdam UMC biobank used for RNA sequencing, patients provided written informed consent. The protocol for retrieval of archival EAC material was in accordance with the Medical Ethical Committee and/or Amsterdam UMC biobank committee of the Amsterdam UMC (AMC 2013_241).

### Histopathological review

Pathology reports and FFPE tissues were obtained for histopathological review. Hematoxylin & Eosin (H&E) stained slides were evaluated to confirm the diagnosis of EAC and to determine the grade of differentiation and to select for high tumor density areas by a dedicated gastrointestinal pathologist, who was unaware of the research and clinical outcomes.

### Targeted sequencing of a gene panel including the SMAD4 gene locus

Targeted sequencing was performed for the SMAD4 gene using an amplicon-based protocol with deep coverage (mean depth 4000x). Genomic DNA was extracted from matched FFPE tissue sections using a QIAamp DNA Mini Kit (Qiagen, Hilden, Germany). Targeted sequencing was performed from 50 ng of genomic DNA for the SMAD4 gene with deep coverage (mean depth 4000x) using a library preparation Lotus DNA Library Prep Kit (IDT, Integrated DNA Technologies, Inc., Coralville, Iowa, USA) and a custom-designed panel IDT2972740 (IDT) for enrichment, according to the manufacturer’s protocol. Libraries were sequenced with a mid-output v2.5 flow cell (300 Cycles) on a NexSeq500 sequencing machine (Illumina). Fastq raw data were mapped, filtered and analyzed according to our internal pipeline (doi 10.1093/brain/awab056). For SMAD4 variant validation, KAPA HiFi HotStart (Roche, Mannheim, Germany) was used for genomic DNA amplification after which the purified PCR products were analyzed by Sanger sequencing according to a previously described method [46].

### Detection of SMAD4 deletion in ISO76A cell line by Sanger sequencing

DNA isolation of the ISO76A cell line was performed using a NucleoSpin®Tissue Kit (BIOKE). A forward primer (5′- GATTTGCGTCAGTGTCATCG-3′) and a reverse primer (5′-GCTGGAGCTATTCCACCTACTG-3′) were used to amplify a certain SMAD4 gene fragment followed by Sanger sequencing.

### Immunohistochemistry

Four μm tissue sections of FFPE tissue blocks were used for IHC. After rehydration, antigen retrieval was performed by incubating the slides for 20 minutes at 98°C in 10 mM sodium citrate buffer at pH 6.0 (BMP4) or 1 mM EDTA pH 9.0 (SMAD4 and SHH). Slides were allowed to cool down and endogenous peroxidases were blocked with Peroxidase Blocking Solution (Sakura Finetek) for 10 minutes at room temperature (RT). Nonspecific sites were blocked with UltraVision Protein Block (Thermo scientific) before primary antibody incubation. All primary antibodies used were diluted in Bright Diluent (VWR) (anti-BMP2 1:200 abcam ab6285 clone 65529.111; anti-BMP4 1:200 abcam ab124715 clone EPR6211; anti-SHH 1:200 abcam ab135240 clone 5H4; anti-SMAD4 1:200 Santa Cruz Biotechnology sc-7966 clone kappa light chain) and incubation was performed over-night at 4°C. Slides were washed in phosphate buffered saline (PBS) and incubated with a BrightVision 1 step detection system and anti-rabbit/mouse secondary antibodies (VWR). The peroxidase activity was visualized using 3,3'-Diaminobenzidine (DAB) chromogen (VWR). Finally, sections were counterstained with Mayer’s hematoxylin (VWR), dehydrated and mounted. The primary antibodies were excluded in the negative controls (PBS). H&E staining was performed in order to correlate the staining pattern against the tissue architecture.

### Interpretation of IHC results

IHC results were scored independently by two researchers who were blinded to the experimental protocol. Scoring was performed semi quantitively for staining intensity (0 if no stain, + if weakly positive, ++ if moderately positive, +++ if strongly positive) and percentage of positive cells (‘1’ if no positive cells, ‘2’ if 1-33% positive cells, ‘3’ if 34-66% positive cells, ‘4’ if 67-100% positive cells). Both stromal and tumor cells were scored. For the final analysis, tumors were divided in low or high expression of BMP2 or BMP4, in case the intensity of the BMP expression was at least ++ in > 3. To define SMAD4 loss, cancer cells showed an intensity of 0 or + in more than 30% of cells, which is in line with previous studies [47].

### Selection of SMAD4 negative cell line from a Tissue Microarray (TMA)

A TMA was established using tissues from six separate EAC PDX lines. These PDXs were established using an intramuscular transplantation technique as previously described [48]. After screening for SMAD4, a SMAD4 negative PDX named ISO76A [48] was identified. A cell line from this PDX (IS076A) was recently established at the Peter MacCallum Cancer Centre, Melbourne, Australia. After splitting, an early passage of this cell line was transfected with Luciferase-eGFP^+^.

### Cell culturing, SMAD4 transfection and cell sorting

SMAD4(-) ISO76A cells were transfected with a SMAD4 expressing construct (ORF expression clone for SMAD4, NM_005359.5, Labomics) and HEK293 cells using Lipofectamine solution (Life Technologies) according to manufacturer’s instructions. To purify ISO76A cells and SMAD4 transfected ISO76A cells from fibroblasts and non-transfected cells, fluorescence-activated cell sorting (FACS) (BD FACSAria™) was performed to select E-cadherin positive and mCherry for sorting SMAD4(+) ISO76A cells, and E-cadherin positive only for sorting SMAD4(-) ISO76A cells. Both SMAD4(-) ISO76A and SMAD4(+) ISO76A cells were cultured in RPMI-1640 culture medium with 10% fetal calf serum (FCS), 2mM glutamine and 100 U penicillin/0.1 mg/ml streptomycin. The cells were placed in an incubator with a 5% CO_2_ concentration at 37°C. All cell lines were tested for Mycoplasma contamination once a month. All in vitro experiments were performed on cells growing exponentially.

### Cell chemoresistance assay

5 × 10^4^ cells/well were seeded in 96-well plates. After 12 hours, the medium was replaced with fresh medium without serum. Next, cells were treated with cisplatin and/or C4C4 and C8C8 for 24 hours at 37°C in a 5% CO_2_ atmosphere. Subsequently, the medium was removed and 100 μl CellTiter-Blue (Promega) solution was added into each well. The 96-well plates were then incubated for an additional 3 hours at 37°C in a 5% CO_2_ atmosphere. Fluorescence was analysed with a Synergy HT Multi-Mode Microplate Reader (Biotek) using 530(25)_Ex_/590(35)_Em_ settings.

### Cell migration assay

10^6^ cells/well were seeded in 6-well plates and grown until 90% confluence. A sterile 200 μl pipette tip was held vertically to scratch (‘wound’) across each well. The detached cells were removed by washing with 2 ml PBS. Cells were treated in triplicate with BMP4, BMP2/4, BMP4 + C4C4, BMP2/4 + C8C8 and control at the specific concentrations for 24 hours. Sample images were obtained at 0 hour and 24 hours using a microscope at 5x magnification. Quantification of wound closure in each well was performed using Image J software.

### Luciferase assay for testing BMP activity

SMAD4(-) ISO76A, OE19, OE33, SK-GT-4, OACM5, OACP4 and Flo-1 cells were cultured in 96-well plates at 5 x 10^3^ cells/well under the same conditions and cells were placed in 37°C incubator overnight to attach. 100 μl RPMI-1640 medium with 0.1% FCS was added to each well. Cells were plated in triplicate. Wells with no cells were used as control. 100 μl luciferase substrate solution from a Bright-Glo Luciferase Assay System (Promega Benelux) was added to each well. After incubation for three minutes at RT, luciferase activity was measured using a Synergy HT Multi-Mode Microplate Reader (Biotek). Normalization of luciferase values was performed by subtracting background activity as measured in the control wells.

### Western blotting

Cells were grown in 6-well plates after which they were washed with PBS and a cell lysis RIPA buffer (Thermo Scientific) containing protease and phosphatase inhibitors (Thermo Scientific) was added. The cells were lysed on ice for 20 minutes, scraped into 1.5 ml tubes, and centrifuged for 15 minutes at 14,000 rcf at 4°C. Protein levels were measured with a BCA kit (Thermo Scientific) using clear lysates. The protein levels in each sample were equalized, the lysates were mixed with 4x loading buffer (Bio-Rad) and boiled at 97°C for five minutes. The proteins were separated by sodium dodecyl sulfate gel electrophoresis (SDS-PAGE) and blotted onto a polyvinylidene difluoride (PVDF) membrane (Millipore, Billerica, MA, USA). The resulting blots were blocked in blocking buffer containing 5% Bovine Serum Albumin (BSA) dissolved in 1x Tris-buffered saline containing 0.1% Tween-20 (TBST) and washed three times in 1x TBST for five min each. Both primary and secondary antibodies were diluted in blocking buffer containing 5% BSA. The primary antibody was incubated overnight at 4°C. A secondary horseradish peroxidase (HRP) linked antibody was incubated for one hour at room temperature after which the blots were exposed to Lumilight+ (Roche, Basel, Switzerland) chemiluminescent substrate and visualized using a chemiluminescence imager (Bio-Rad).

### Mice

Animal experiments were performed in accordance with the National Health and Medical Research Council Australian Code of Practice for the Care and Use of Animals for Scientific Purposes and approved by the Peter MacCallum Cancer Centre (PMCC) Animal Experimentation Ethics Committee. NOD-SCID IL-2Rγ^KO^ (NSG) mice were bred in-house.

### Tumor xenografts

To establish xenografts of the EAC cell line ISO76A (PDX model) transfected with e-GFP, 2.5 million cells suspended in 100 μl 1:1 PBS and Matrigel (BD Bioscience) were subcutaneously injected into the flank of 6-8 weeks female immunodeficient NSG mice. Subcutaneous tumor volumes were determined biweekly with calipers and calculated using the formula (length × width^2^)/2. All mice were euthanized when subcutaneous tumors reached ≥ 1500 mm^3^ or if humane endpoints were reached (labored breathing, bloated abdomen or weight loss in excess of 15% of baseline body weight).

### In vivo bioluminescence imaging of PDX in NSG mice

To assess the establishment and growth of the PDX in the mice, in vivo imaging was performed. Luciferase-eGFP^+^ ISO76A cells (2.5 million cells per mouse) suspended in PBS and Matrigel were subcutaneously injected into NSG mice. Animals were imaged on a Xenogen IVIS 100 Imaging System (Caliper Life Science) for life imaging of the tumor xenografts. 100 μl of 20 mg/ml luciferin (Promega) in PBS was subcutaneously injected into each mouse 5 min before imaging. Imaging was performed under general anesthesia. Animals were shaved before imaging. Live imaging was performed at different endpoints and at the end of the study. At the study endpoint, the whole mouse and its organs were imaged to determine the tumor burden. The imaging exposure times were 60 s for whole animals and 5 min for organs. The bioluminescence signal was quantified using Living Image software.

### In vivo imaging of BMP2/4 expression and delivery of VHHs in the PDX model

To assess the biodistribution of our recently developed highly specific VHHs (C4C4 and C8C8) within the PDX model, in vivo imaging was performed. To this end, C4C4 and C8C8 labeled with IRDye800cw (QvQ, Utrecht, the Netherlands) was used. At approximately two weeks post inoculation, three mice bearing tumors with a volume in excess of 1000 mm^3^ were used to investigate the delivery and retention of C4C4 and C8C8 within the tumor xenografts. Mice were administered 25-50 μg of labeled VHHs through tail vein injection and imaged after 30 minutes, 2 hours, 4 hours and 24 hours. Animals were imaged on a Xenogen IVIS 100 Imaging System (Caliper Life Science). At the end of the experiment, mice were sacrificed and their organs imaged to determine the retention of the drug in various organs in addition to the tumor xenograft. The imaging exposure times were 60 seconds for the whole animal and up to 5 minutes for ex vivo imaging of the organs. The infrared signal was quantified using the ‘region of interest’ function in Living Image software.

### Treatment of the PDX model with C4C4 and C8C8

To test the anti-tumor activity of C4C4 and C8C8, both individually and in combination with cisplatin, xenografts were established subcutaneously in 30 NSG mice using firefly-luciferase-expressing SMAD4(-) ISO76A cells as previously described. The tumor growth was evaluated twice per week using electronic calipers. For treatment, animals were randomized into six groups: group 1 received vehicle (0.9% NaCl); group 2 received cisplatin 2 mg/kg once per week; group 3 received daily 100 μg C4C4 via intraperitoneal injection (IP); group 4 received daily 100 μg C8C8 IP; group 5 received cisplatin 2 mg/kg once per week in combination with C4C4, 100 μg daily IP, and group 6 received cisplatin 2 mg/kg once per week and C8C8, 100 μg daily IP. Treatment was started once the PDX reached a volume of at least 80 mm^3^ and continued for 4 weeks or until tumors reached a volume of 1500 mm^3^ or a humane endpoint was reached. After reaching the endpoints the mice were sacrificed and organs were harvested.

### Survival study of SMAD4(-) ISO76A based PDTX model with continued treatment of C4C4 and C8C8

To evaluate long term outcomes, a survival study was performed using the same SMAD4(-) ISO76A PDX model. After an induction treatment for 4 weeks with C4C4, C8C8 and cisplatin (2 mg/kg), C4C4 and C8C8 treatment regimens were continued in doses of 100 μg five times per week, 100 μg three times per week and 100 μg once per week, in combination with cisplatin (2 mg/kg), until humane endpoints were reached. Control groups were animals treated with cisplatin (2 mg/kg) once per week for 4 weeks and animals treated with the vehicle (0.9% NaCl). Mice were euthanized when the PDX reached a volume of ≥ 1500 mm^3^ or when the mice showed signs of ill health or a weight loss of > 15% of their baseline body weight.

### Statistical analysis

All data consolidation, organization and processing were carried out using Microsoft Excel. Image J was used for quantifying gray levels of bands in Western blotting and cell wound closure levels in cell migration assays. GraphPad Prism 9 was used for testing significant differences for cell chemoresistance and tumor volumes by paired *t*-test, and survival analysis by Log-rank (Mantel-Cox) test. For the remaining experiments, unpaired *t*-test and two-tailed distribution at the 5% level were used. IBM SPSS was used for performing survival analyses through the Kaplan Meier method.

## Results

### Analysis of public databases indicates a phenotype of SMAD4 mutated cancers with dysregulation of non-canonical pathways

**Fig. 1:**
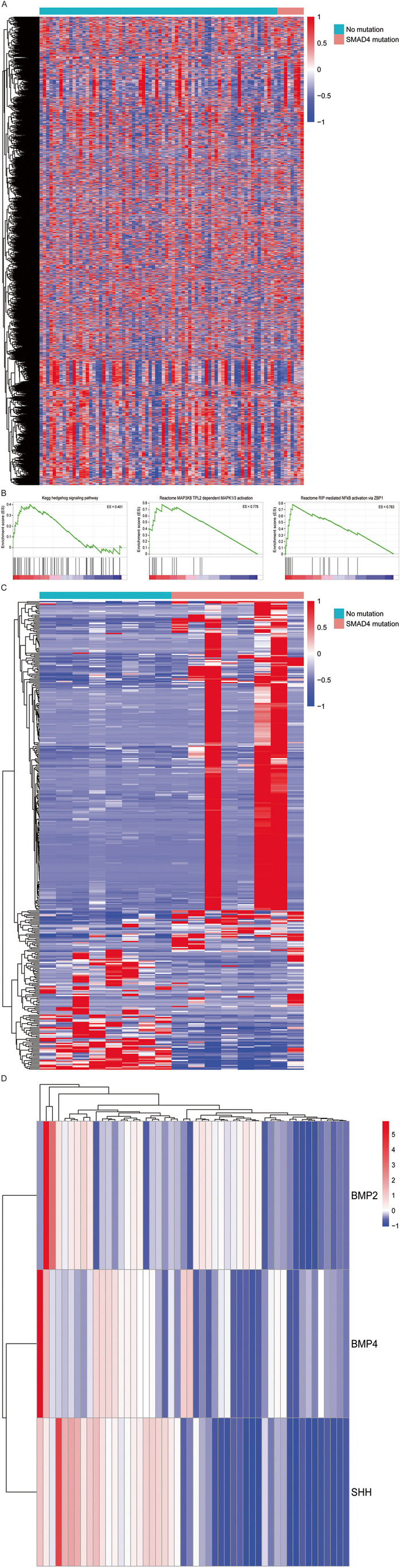
(**A**) The 5000 most variable gene expression profiles of 80 EAC cases between SMAD4 mutated and non-mutated EAC cases. (**B**) GSEA enrichment plots for differentially expressed pathways of interest. (**C**) Comparison of the 8 SMAD4 mutated cases versus 8 SMAD4 non-mutated with highest SMAD4 expression, indicating 382 significantly differetially expressed genes. (**D**) Expression of SHH, BMP2 and BMP4 in a cohort of 50 EAC cases analysed by RNA sequencing

From the TCGA dataset, count data and SMAD4 mutation annotation data were available from 80 patients with EAC. Of these 80 patients, eight patients had somatic mutations in SMAD4, including five missense mutations and three nonsense (point) mutations. The other 72 patients had no somatic mutations in SMAD4. The expression data of these 80 patients with annotation for SMAD4 mutation status is shown in a heatmap (Fig. 1A). The expression data with annotation were used as the source of input for GSEA. The results of this analysis are shown in Supplementary File 1. In summary, 54 pathways were significantly enriched in the SMAD4 mutated EAC samples from the REACTOME database, with the MAP3K8_TPL2_DEPENDENT_MAPK1_3_ACTIVATION being identified as the most significantly enriched REACTOME pathway. In addition, 11 pathways were identified from the KEGG database, including the KEGG Hedgehog signaling pathway. Seven pathways were identified from the BIOCARTA database (Fig. 1B). In sum, the GSEA analyses confirmed dysregulation of several oncogenic pathways in the SMAD4 mutated EAC samples.

To investigate more closely which dysregulated pathways are associated with differences in SMAD4 mutation status and expression, eight patients with somatic SMAD4 mutations were compared to 8 patients with the highest SMAD4 expression by differential expression analysis. This analysis resulted in 382 genes being significantly differentially expressed, among which were SMAD4 and SMAD2, which both exhibited a low expression in SMAD4 mutated samples. Results of the differential expression analysis can be found in Supplementary File 2. A heatmap of these 16 samples and the 382 differentially expressed genes is shown in Fig. 1C.

Inputting the list of 382 differentially expressed genes into IPA resulted in 10 IPA pathways being significantly differentially expressed. All 10 pathways were significantly activated in EAC samples without SMAD4 mutation. Among these 10 pathways is the p38 MAPK pathway. Within this pathway, a number of differentially expressed genes were identified. These included IL1A, IL1RN, IL36A, IL36B, IL36G, IL36RN and PLA2G4E, which all exhibited high expression levels within SMAD4 mutated samples. In contrast MAPT was found to exhibit a low expression in SMAD4 mutated samples. These pathways and their z-scores are shown in Table 1. The complete output of the IPA analysis can be found in Supplementary File 3.

**Table 1:** Significantly differentially activated IPA pathways. A z-score above 2 means that the pathway is activated in EAC samples without SMAD4 mutation compared to EAC samples with a somatic SMAD4 mutation

Ingenuity Canonical Pathways	-log(*p*-value)	Ratio	Z-score	Molecules
Nicotine Degradation II	2.03	0.06	2.00	ADH7,CYP2E1,CYP4B1,FMO2
IL-6 Signaling	2.26	0.05	2.45	IL1A,IL1RN,IL36A,IL36B,IL36G,IL36RN
Cholecystokinin/Gastrin-mediated Signaling	2.41	0.05	2.45	IL1A,IL1RN,IL36A,IL36B,IL36G,IL36RN
Adrenomedullin Signaling Pathway	3.02	0.05	2.83	CALML5,GNA15,IL1A,IL1RN,IL36A,IL36B,IL36G,IL36RN,SOX15
Role of Hypercytokinemia/Hyperchemokinemia in the Pathogenesis of Influenza	3.13	0.07	2.45	IL1A,IL1RN,IL36A,IL36B,IL36G,IL36RN
Acute Phase Response Signaling	3.25	0.05	3.00	FGA,FGB,FGG,IL1A,IL1RN,IL36A,IL36B,IL36G,IL36RN
Toll-like Receptor Signaling	3.36	0.08	2.45	IL1A,IL1RN,IL36A,IL36B,IL36G,IL36RN
p38 MAPK Signaling	3.91	0.07	2.65	IL1A,IL1RN,IL36A,IL36B,IL36G,IL36RN,MAPT,PLA2G4E
Intrinsic Prothrombin Activation Pathway	4.88	0.14	2.45	FGA,FGB,FGG,KLK12,KLK13,KLK8
Neuroprotective Role of THOP1 in Alzheimer's Disease	7.51	0.10	2.33	ENDOU,HPN,KLK12,KLK8,MAPT,PRSS27,SRY,TMPRSS11A,TMPRSS11B,TMPRSS11D,TMPRSS11E,TPSD1

### SMAD4 and BMP2/4 expression with respect to patient outcomes

**Fig. 2:**
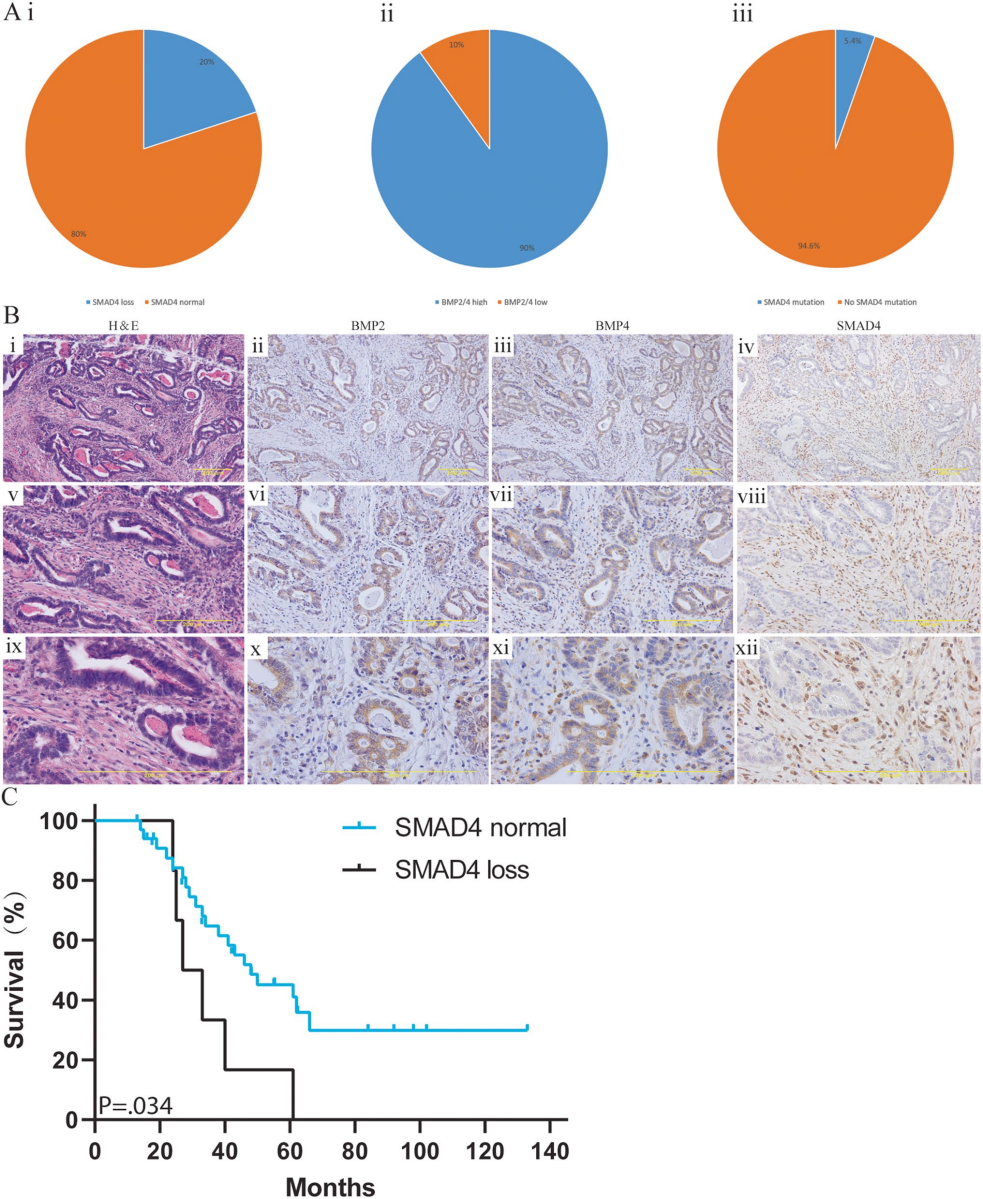
(**Ai**) SMAD4 expression by IHC. (**Aii**) BMP2 and 4 expression by IHC. (**Aiii**) SMAD4 loss determined by targeted sequencing. (**B**) Lanes from left to right: H&E staining, IHC for BMP2, BMP4, and SMAD4, of an EAC patient indicating high BMP2/4 expression and SMAD4 loss in tumor cells but not in the stromal tissue. (**C**) Kaplan–Meier curve depicting overall survival stratified for patients with high and low expression of SMAD4 (log rank test, *p* < 0.05)

All EAC patients from our cohort were diagnosed with EAC between 2005 and 2009 and treated by esophageal resection at the Amsterdam UMC. None of the patients received adjuvant or neoadjuvant therapies. Of the 40 patients, 32 were male and the median age was 62 (range 41-81). All cases were stage 3 and 4 tumors (Supplementary Table 1, all patients with stage 4 disease were classified as such because of suspicious lymph nodes at the truncus coeliacus, but were considered to be operable) according to the AJCC classification. Patient characteristics and IHC results are listed in Supplementary Table 1. Using a cut-off of > 33 % positive cells with an intensity score of ++ or higher by IHC, we found that 90% of cases showed increased BMP2 and/or BMP4 expression levels (Fig. 2A). Since BMP2/4 can induce non-canonical signaling, in case of corrupted canonical BMP signaling through SMAD4 loss, we analysed the SMAD4 status by IHC (Fig. 2B) and targeted sequencing. 20% (8/40) of our cases harboured at least 30% of cells with loss of SMAD4 expression by IHC, while SMAD4 mutations were found in 5% (2/37) of the cases (Fig. 2A). In the mutated cases, the gene mutational status was concordant with the IHC results. Cases with loss of SMAD4 expression were also shown to have a poorer disease-free survival (*p* < 0.05, Fig. 2C). All cases with loss of SMAD4 expression showed high expression levels of BMP2 and/or 4. Together, these results indicate that SMAD4 mutations and loss of SMAD4 expression occur in a subset of EAC cases with poorer clinical outcomes. It seems that cases with SMAD4 loss have high BMP2 and/or 4 expression levels, which may trigger non-canonical BMP signaling via oncogenic pathways.

### Selection of a SMAD4(-) ISO76A cell line and generation of SMAD4(+) ISO76A cell lines

**Fig. 3:**
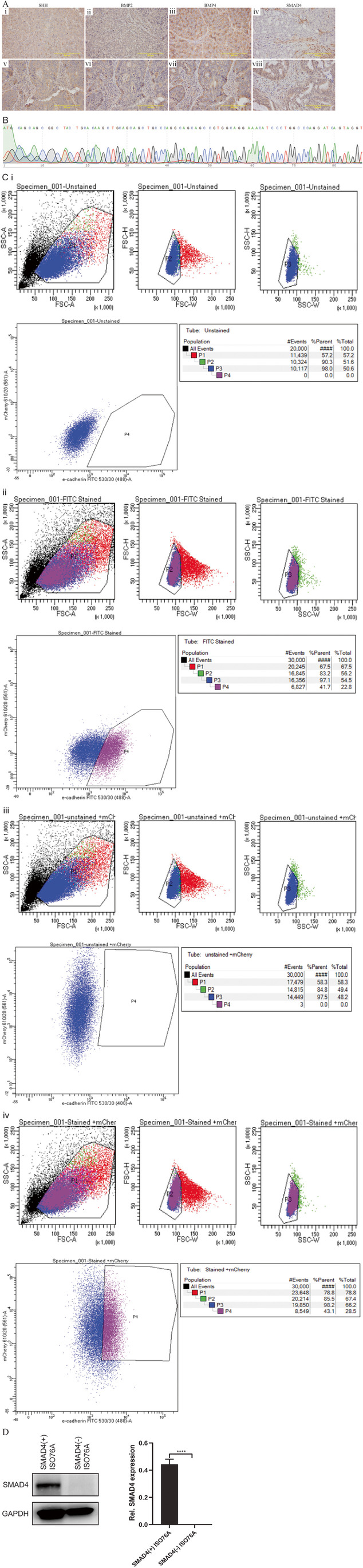
(**A**) IHC for SHH, BMP2, BMP4 and SMAD4 of ISO76A (i, ii, iii, iv) and a SMAD4 positive EAC (v, vi, vii, viii). (**B**) SMAD4 gene deletion of ISO76A cell line determined by Sanger sequencing. (**C**) FACS analysis for isolation of pure populations of SMAD4(-) ISO76A and mCherry expressing SMAD4(+) transfected ISO76A cells using E-cadherin-FITC labeling of cells. (i) Unstained SMAD4(-) ISO76A cells as negative control. (ii) Gating of FITC labeled E-cadherin expressing SMAD4(-) ISO76A tumor cells. (iii) mCherry expressing SMAD4(+) transfected ISO76A cells as negative control. (iv) Isolation of mCherry positive and FITC labeled E-cadherin expressing SMAD4(+) ISO76A cells. (**D**) Western blot analysis for SMAD4 in SMAD4(-) ISO76A and SMAD4(+) ISO76A cell lines. Relative gray values normalized to GAPDH levels are indicated below the corresponding bands. GAPDH served as a loading control

The currently available commercial EAC cell lines OE19, OE33, Flo-1, SKGT4, OACM5 and OACP4 all exhibit a normal gene status and expression level of SMAD4. To select an EAC cell line with a loss of SMAD4, IHC for SMAD4 was performed on a TMA containing PDXs established from primary cell lines of 6 EAC patients. Absence of SMAD4 expression was found only in the cell line ISO76A, with normal SMAD4 expression in the remaining six cell lines (Fig. 3A). IHC also indicated that the PDXs established from ISO76A had retained expression of SHH, BMP2 and BMP4 (Fig. 3A). Sequence analysis confirmed a SMAD4 deletion (AGGC) in ISO76A (Fig. 3B). The SMAD4(-) ISO76A cells were successfully transfected with a SMAD4 gene construct labelled with mCherry. By FACS analysis, SMAD4(+) ISO76A cells were successfully purified from untransfected cells and fibroblasts through selecting E-cadherin and mCherry positive cells (Fig. 3C). Likewise, SMAD4(-) ISO76A cells were purified from fibroblasts by selecting only E-cadherin positive cells (Fig. 3C). The expression of SMAD4 in SMAD4(+) ISO76A and SMAD4(-) ISO76A cells was validated by Western blotting (Fig. 3D).

### BMP expression is decreased in cultured EAC cell lines, but increased in PDXs

**Fig. 4:**
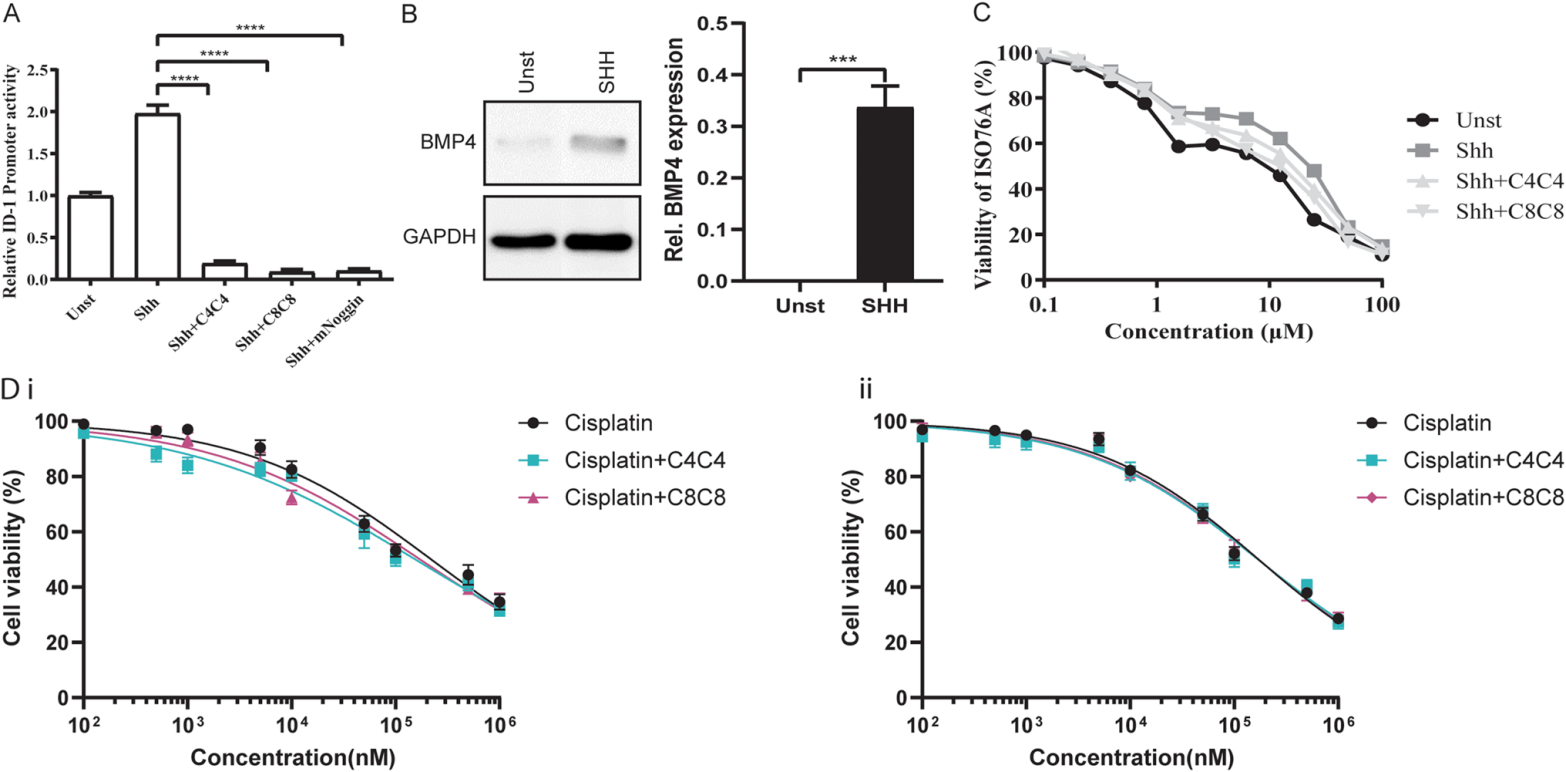
(**A**) Luciferase analysis of BMP activity in SMAD4(-) ISO76A cells stimulated by SHH (1 μg/ml) and upon inhibition with C8C8 (500 ng/ml), C4C4 (500 ng/ml) and Noggin (500 ng/ml) for 16 hours (*p* < 0.01). Results are relative to the baseline level of the medium. (**B**) Western blot analysis of BMP4 expression in SMAD4(-) ISO76A cells treated with SHH (1 μg/ml). Relative gray values normalized to GAPDH levels are indicated below the corresponding bands. GAPDH served as a loading control. (**C**) Cell viability assay of in SMAD4(-) ISO76A cells stimulated by SHH (1 μg/ml) and inhibited by C8C8 (500 ng/ml), C4C4 (500 ng/ml) and Noggin (500 ng/ml), respectively, for 16 hours (*p* < 0.01). (**D**) Cell viability assay of SMAD4(-) ISO76A (i) (*p* < 0.01) and SMAD4(+) ISO76A (ii) (*p* > 0.05) stimulated by cisplatin and inhibited by C8C8 and C4C4. The Y-axis indicates the relative cell viability normalized to the untreated control

BMP secretion as measured by the luciferase assay was low in ISO76A cells and in the commercial EAC cell lines: Flo-1, OE33, SKGT4, OACM5 and OACP4. Only OE19 had maintained secretion of BMP under normal culture conditions (Supplementary Fig. 1). The low BMP secretion is most likely due to the absence of stimulating upstream ligands, such as SHH, which are normally secreted by cells in the microenvironment or by subsets of cancer cells [12]. From the analysis of the expression profiles, we found SHH to be highly expressed in a subset of EAC. High SHH expression also significantly correlated with high BMP2 (Spearman rank correlation test; *p*-value = 0.01301, Spearman’s correlation coefficient 0.3489243) and BMP4 (Spearman rank correlation test; *p*-value = 0.0006267, Spearman’s correlation coefficient 0.4671053) expression (Fig. 1D). IHC analysis of the TMA containing a PDX of the ISO76A cell line revealed that subsets of cancer cells within the PDX express SHH (Fig. 3A). As measured by the luciferase assay, stimulation of cultured SMAD4(-) ISO76A cells with SHH induced BMP activity (Fig. 4A), which could be effectively inhibited by the natural BMP antagonist Noggin (Fig. 4A). To investigate whether and to what extent the BMP activity was derived from the expression of BMP2 and 4, we incubated the cells with C4C4 and C8C8, which are highly specific inhibitors of BMP4 and BMP2/4, respectively [36]. We found that inhibition of BMP4 alone by C4C4 already significantly reduced BMP activity, whereas inhibition of BMP2/4 by C8C8 resulted in a nearly complete inhibition of the BMP activity (Fig. 4A). These results indicate that SHH upregulates BMP expression and activity in SMAD4(-) ISO76A cells and that this activity is mostly due to the expression of BMP2 and 4. Using Western blotting, we confirmed that SHH upregulated the expression of BMP4 in SMAD4(-) ISO76A cells (Fig. 4B).

### In vitro inhibition of BMP2/4 decreases chemoresistance of SMAD4(-) ISO76A cells to cisplatin

To investigate whether elevated BMP2 and 4 expression affect chemoresistance in EAC, and to compare the effects of BMP4 and BMP2/4 inhibition in chemoresistance in the SMAD4(+) ISO76A and SMAD4(-) ISO76A cells, we performed cell viability assays. Both the SMAD4(+) ISO76A and SMAD4(-) ISO76A cells were stimulated with cisplatin with and without C4C4 and C8C8. Inhibition of BMP4 and BMP2/4 in the SMAD4(+) ISO76A cells did not show any significant difference in resistance to cisplatin. In contrast, C4C4 and C8C8 both significantly decreased the resistance of SMAD4(-) ISO76A cells (*p* < 0.01, Fig. 4D), with IC50 values of SMAD4(-) ISO76A compared to SMAD4(+) ISO76A (Supplementary Table 2). Thus, the chemoresistance to cisplatin of the SMAD4(-) ISO76A cells could be significantly decreased by inhibiting BMP4 and BMP2/4 using C4C4 and C8C8, respectively. Additionally, we found that SHH stimulation increased the viability of SMAD4(-) ISO76A cells, which could be rescued by C4C4 and C8C8 (*p* < 0.05, Fig. 4C). This indicates again that SHH, as an upstream ligand, can upregulate the expression of BMP2 and 4. This effect can be inhibited through the use of the VHHs.

### In vitro inhibition of BMP2/4 impairs the migration of SMAD4(-) ISO76A cells

**Fig. 5:**
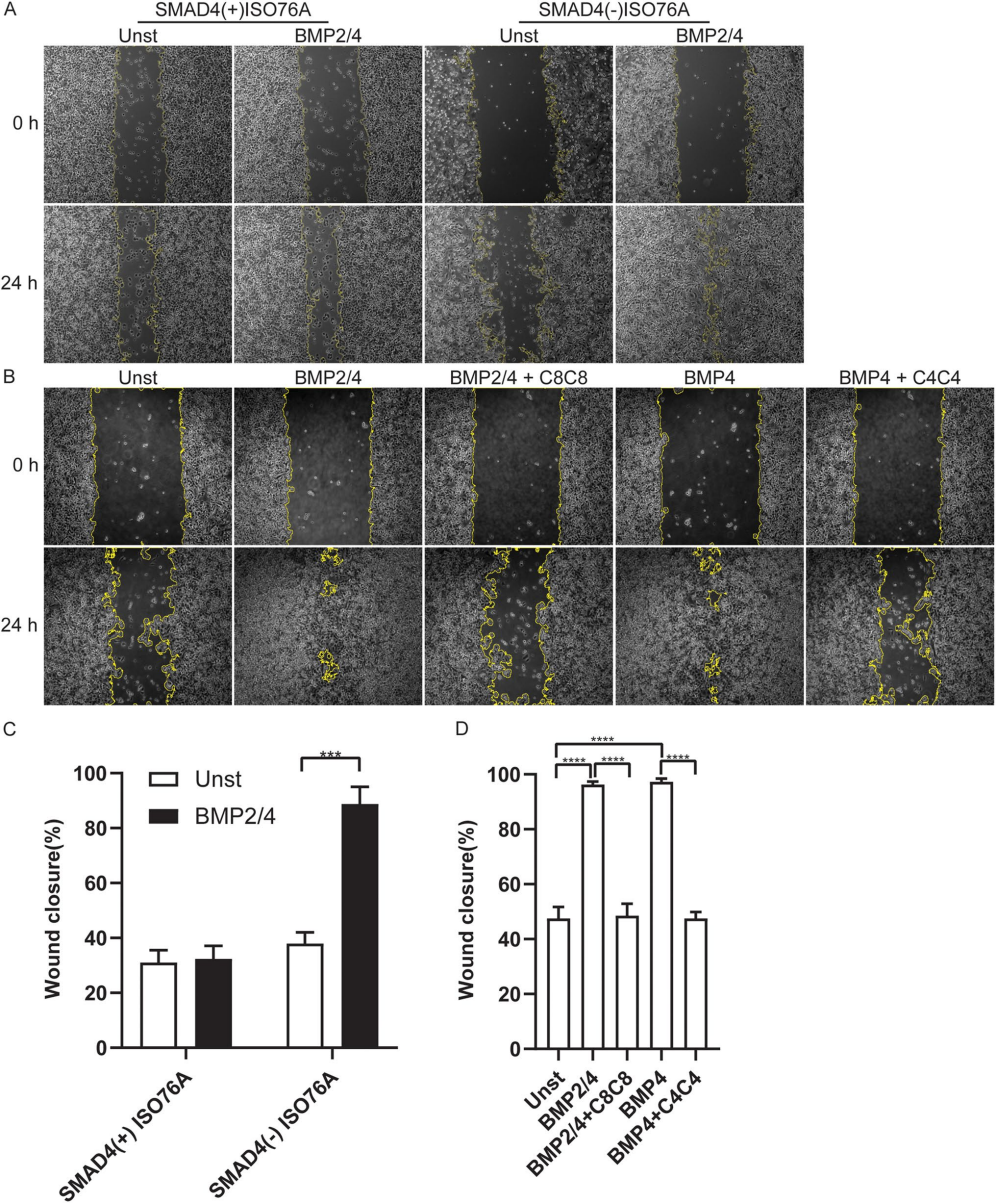
(**A**) Migration analysis for SMAD4(-) ISO76A and SMAD4(+) ISO76A cells using a scratch wound healing cell migration assay. A scratch was made in 90% confluent cultured SMAD4(+) ISO76A and SMAD4(-) ISO76A cells. Next, the cells were stimulated by BMP2/4 (each 100 ng/ml) for 24 h. (**B**) Scratch wound healing assay of SMAD4(-) ISO76A cells stimulated by BMP2/4 (100 ng/ml) and/or BMP4 (100 ng/ml) with or without inhibition by C8C8 (500 ng/ml) and C4C4 (500 ng/ml) for 24 h. (**C**) Quantification of wound closure of ISO76A cells with and without BMP2/4 stimulation (*p* < 0.01). (**D**) Quantification of wound closure of SMAD4(-) ISO76A cells stimulated by BMP2/4 and/or BMP4 with or without inhibition by C8C8 and C4C4 (*p* < 0.01)

To study whether BMP2 and/or BMP4 have an effect on the migration of SMAD4(-) ISO76A and SMAD4(+) ISO76A cells, a scratch wound healing assay was performed. First, both the SMAD4(+) ISO76A and SMAD4(-) ISO76A cells were stimulated with BMP2/4, which revealed no significant difference between the BMP2/4 stimulated and control groups for the SMAD4(+) ISO76A cells. In contrast, for the SMAD4(-) ISO76A cells, BMP2/4 stimulation showed a significant faster wound closure compared to the control group (*p* < 0.01, Fig. 5A and C ). Thus BMP2/4 has a significant effect on the migration of SMAD4(-) ISO76A cells, but not on that of SMAD4(+) ISO76A cells. To assess whether C4C4 and C8C8 can inhibit migration, we stimulated SMAD4(-) ISO76A cells with BMP4 and BMP2/4, and treated them with the BMP inhibitors C4C4 and C8C8, respectively. The results of BMP2/4 stimulated cells were consistent with the previous experimental results. Furthermore, we found that BMP4 also stimulated wound closure and that this could be significantly inhibited by C4C4 (*p* < 0.01, Fig. 5B and D). Similar results were obtained for the cells stimulated with BMP2/4 and treated with C8C8 (*p* < 0.01, Fig. 5B and D). These data suggest that BMP2 and BMP4 can increase the migration of SMAD4(-) ISO76A cells but not of SMAD4(+) ISO76A cells. These results also indicate that C4C4 and C8C8 can effectively inhibit BMP2/4 or BMP4 induced migration of SMAD4(-) ISO76A cells.

### BMP2/4 induce non canonical signaling but not EMT in SMAD4(-) ISO76A cells

**Fig. 6:**
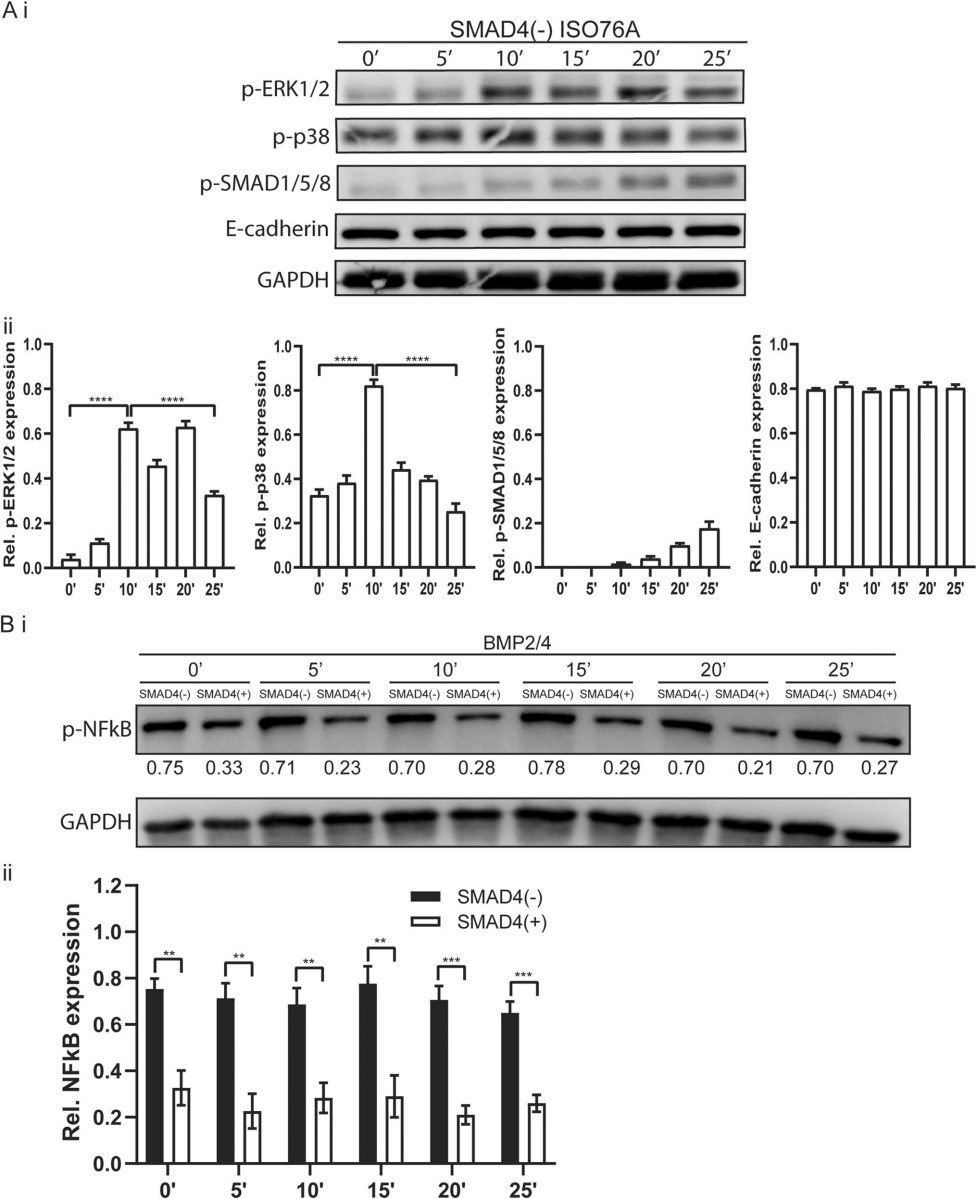
(**Ai**) pSMAD1/5/8, pERK1/2, p38 and E-cadherin levels in SMAD4(-) ISO76A cells stimulated by BMP2/4 (BMP2 (100 ng/ml) or BMP4 (100 ng/ml)) for 0’, 5’, 10’, 15’, 20’ and 25’. (**Aii**) Quantification of outputs as observed in (Ai). GAPDH served as loading control. (**Bi**) pNFk-B levels in SMAD4(+) ISO76A and SMAD4(-) ISO76A cells stimulated by BMP2/4 (each 100 ng/ml) for 0’, 5’, 10’, 15’, 20’ and 25’. (**Bii**) Quantification of outputs as observed in (Bi). GAPDH served as loading control

One of the previously described mechanisms of malignant BMP signalling in SMAD4 negative cancers is through increased epithelial-mesenchymal-transition (EMT) [49]. We analysed the effects of BMP2 and BMP4 on EMT and BMP canonical and non-canonical signalling. BMP2/4 stimulation induced strong canonical signalling as observed by the increased expression of pSMAD1/5/8 (pSMAD) in the SMAD4(+) ISO76A cells (Supplementary Fig. 2), while SMAD4(-) ISO76A cells showed minimal pSMAD expression (Fig. 6A). Western blot analysis of the EMT markers showed that BMP stimulation did not change EMT expression in the SMAD4(-) ISO76A cells (Fig. 6A), nor was there any change of these markers observed in the SMAD4(+) ISO76A cells (Supplementary Fig. 2). BMP2/4 can upregulate signaling via alternative pathways such as the p38 MAPK pathway, as we described earlier [35]. Here, we found that in SMAD4(-) ISO76A cells, stimulation with BMP2/4 increased the p38 MAPK pathway, while there was minimal BMP canonical signaling (Fig. 6A). In contrast, in the SMAD4(+) ISO76A cells, stimulation with BMP2/4 increased pSMAD expression, while there was a minimal change within the p38 MAPK pathway (Supplementary Fig. 2). Thus, it seems that the malignant behaviour of SMAD4(-) ISO76A cells can at least in part be explained by BMP2/4 non-canonical p38 MAPK signaling, while EMT effects are not observed. To study whether there is any difference between BMP2 and BMP4 stimulation, we stimulated SMAD4(-) ISO76A cells with BMP2 and BMP4, separately. We found that there was no significant difference between BMP2 and BMP4 stimulation for SMAD4(-) ISO76A cells (Supplementary Fig. 3). Furthermore, we found that BMP2/4 stimulation did not affect the expression of p-NFkB in SMAD4(-) ISO76A cells, nor in SMAD4(+) ISO76A cells, although the expression of p-NFkB in SMAD4(-) ISO76A cells was higher than that in SMAD4(+) ISO76A cells (Fig. 6B).

### Bioluminescence and targeted imaging of VHHs in the PDX model

**Fig. 7:**
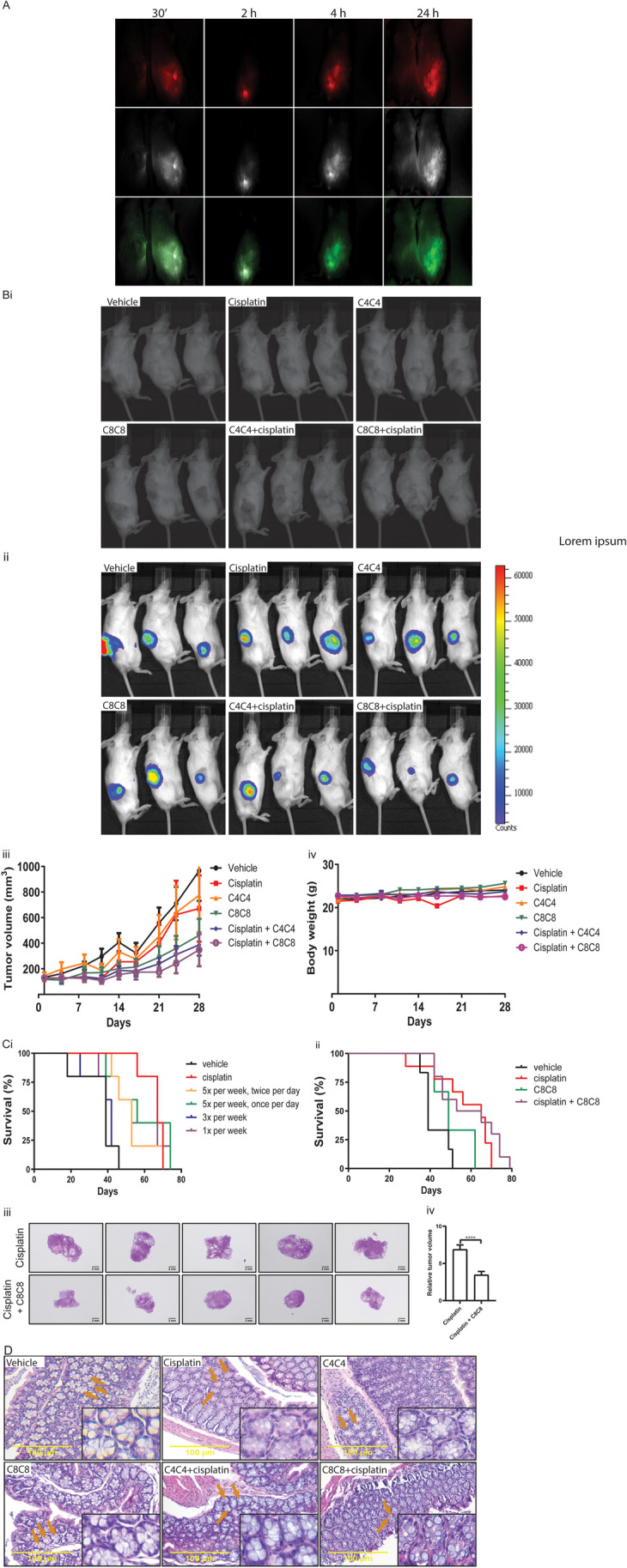
(**A**) Selective targeting BMP4 and BMP2/4 using IRDye800cw labeled C4C4 and C8C8 in a PDX NSG mouse model established by SMAD4(-) ISO76A at 28 days following treatment. Imaging at 30’, 2 h, 4 h and 24 h following intravenous injection with 50 ng IRDye800cw-labeled C4C4 and C8C8. (**Bi** and **Bii**) Normal white light imaging and relative luciferase activity in the (Luciferase-eGFP-SMAD4(-) ISO76A) PDXs in NSG mice of each treatment arm at 28 days following treatment. Representative mice are shown. (**Biii**) Tumor growth curves in mice 28 days following subcutaneous injection of 2.5 million cells into the right flank of NSG mice treated with 0.9% NaCl saline, cisplatin (2 mg/kg), C4C4 (100 μg/day), C8C8 (100 μg/day), C4C4+cisplatin and C8C8+cisplatin (*p* < 0.05). (**Biv**) Weight curves of mice during 28 days following subcutaneous injection of 2.5 million cells into the right flanks and treatments (*p* > 0.05). (**Ci** and **Cii**) Survival curves of mice from each group (*p* < 0.05). (**Ciii**) H&E staining of PDXs of the cisplatin compared to the cisplatin + C8C8 group indicating the largest cross-sectional areas following the survival experiments. (**Civ**) Quantification of largest cross sectional areas of cisplatin and cisplatin + C8C8 groups (*p* < 0.01). (**D**) Analysis for side effects of cisplatin and VHHs on the intestine of mice colons. H&E staining for colon tissues from six treatment groups at 28 days following treatment

To evaluate the effects of BMP inhibition in vivo, we first tested whether the PDXs of SMAD4(-) ISO76A cells as established in NSG mice re-expressed BMP2/4. The expression of BMP2/4 in the PDX was assessed using infrared (IRDye800cw) labeled C4C4 and C8C8. After intravenous injection of the animals with the IRDye800cw labeled VHHs, secretion of the VHHs via the kidney and bladder could be observed two hours post injection. After four hours, retention of the labeled VHHs in the tumors was visible and the labeled VHHs were still retained in the PDXs 24 hours after injection (Fig. 7A). Ex vivo imaging of the mouse organs confirmed physiological retention of the VHHs in the liver and kidneys, which metabolize and secrete the VHHs, and pathological retention in the PDXs (Fig. 7A). These results not only show that BMP2/4 are re-expressed in the PDXs, but also that C4C4 and C8C8 effectively target BMP4 and BMP2/4, respectively, and are retained in ISO76A tumor cells in the PDX model.

### VHHs impair tumor growth in the PDX model

To further investigate whether inhibition of BMP4 and BMP2/4 in SMAD4(-) ISO76A cells could attenuate tumor growth and potentiate the effect of cisplatin in the PDX model, we compared the effects of C8C8 and C4C4 with and without cisplatin treatment in vivo. In vivo imaging of the PDXs was possible since the cells carried a Luciferase-eGFP^+^ construct. The PDXs could be visualized through bioluminescence. PDX formation in the flank of the mice could already be observed two weeks after subcutaneous inoculation with Luciferase-eGFP^+^ SMAD4(-) ISO76A cells, while a tumor volume of 1500 mm^3^ was reached after a median period of eight weeks. At day 21 we could already observe that both C4C4 and C8C8, when given as single agents, inhibited tumor growth compared to the saline control animals. In combination with cisplatin, these anti-tumor effects were further potentiated. After 28 days, the differences between tumor growth between the groups were even more significant (*p* < 0.05, Fig. 7Biii and Supplementary Fig. 4). In vivo treatment was also well tolerated with mice maintaining their body weight (*p* > 0.05, Fig. 7Biv). The measured tumor values corresponded with the results obtained by luciferase bioluminescence (Fig. 7Bi and Bii). These results indicate that targeting BMP2/4 in SMAD4(-) ISO76A cells inhibits in vivo tumor growth and acts synergistically with cisplatin. Thus targeting BMP2/4 in an in vivo PDX model of SMAD4(-) EAC cells decreases chemoresistance and induces tumor cell death.

### VHHs improve the survival and decrease side effect of cisplatin on colon cancer cells in the PDX model

To evaluate the effects of the BMP inhibitors over a longer period of time, after an induction treatment with cisplatin and C8C8 or C4C4, treatment with the VHHs was continued in different dosages. We found that mice that were treated continuously with C8C8 at a dosage of 100 μg five times per week exhibited a significantly longer survival before humane endpoints were reached when compared to mice in the vehicle group (*p* < 0.05, Fig. 7Ci). C8C8 in a dosage of 100 μg five times per week synergistically increased the survival time with cisplatin (*p* < 0.05, Fig. 7Cii). Ex vivo analysis of the PDTXs confirmed smaller tumor sizes in the animals with continued treatment with C8C8 (*p* < 0.01, Fig. 7Ciii and Civ). As a sign of the toxic effect of cisplatin on the gastrointestinal tract, we only found that the goblet cells within the colonic epithelium of the mice were reduced (Fig. 7D). Interestingly, C4C4 and C8C8 did not affect the goblet cells compared to the cisplatin group (Fig. 7D). Above all, when cisplatin was combined with VHHs, the gastrointestinal side effects were not seen (Fig. 7D).

### Expression of BMP2, BMP4, SMAD4 and SHH in the PDX model

To analyse the expression of proteins of interest in the PDX model, we performed IHC staining for BMP2, BMP4, SMAD4 and SHH on tumor, lung and colon tissue 28 days post treatment. We found that BMP2, BMP4 and SHH were highly expressed in tumor, lung and colon (Supplementary Fig. 5A). SHH was not only highly expressed in tumor cells, but also in many stroma cells (Supplementary Fig. 5A), which could be the reason why BMP expression is high in EAC tumor tissues but low in EAC cell lines. SMAD4 remained negative in tumor cells. In contrast, normal expression of SMAD4 was observed in lung and colon tissues (Supplementary Fig. 5B and C).

## Discussion

EACs are genetically highly instable, and characterized by a high mutational load [50]. Due to the heterogeneity of these cancers, response to the current standard care of treatment is unpredictable, whereas the overall 5 year survival outcomes are poor. Identifying subsets of patients who can benefit from targeted therapies is, therefore, of paramount importance for improving EAC patient outcomes. The SMAD4 gene has been found to be frequently mutated in a subset of EAC [8, 51]. Within the SHH-BMP-SMAD signaling cascade, SMAD4 is a downstream intracellular target, which together with several nuclear factors, regulates the transcription of factors involved in cell growth and development. Derangement of this signaling cascade through SMAD4 gene mutations can lead to alternative SHH-BMP signaling via non-canonical/oncological routes. Previous studies have shown that BMP signaling in the absence of SMAD4 leads to the activation of a broad range of non-canonical signaling pathways and to enhancement of invasion and metastasis in colorectal cancer [7]. Our aim was to gain more insight into the biological mechanisms and to develop a targeted therapy for EACs that carry SMAD4 mutations and/or deletions. The mutational rate of this gene is generally found between 8-10% in EAC and to be associated with more frequent disease recurrences and a poor survival [8–10]. We first analyzed a cohort of in house EAC cases and cases from the TCGA database for SMAD4 mutations, which confirmed the mutational rate to be between 5 and 10%. However, by IHC, we found decreased expression of SMAD4 in 20% of our cases and that this loss of SMAD4 expression was significantly correlated with poor survival. Discordance between SMAD4 mutational status and protein expression has been described earlier and could be due to epigenetic silencing mechanisms regulating gene expression [47]. The finding of a higher number of patients with loss of SMAD4 expression, however, is of importance for the future selection of patients. It could mean that potentially more patients may benefit from treatments targeting tumors with a deficient SMAD4 status.

We found that all of the SMAD4 negative EAC cases exhibited preserved expression of BMP2 and/or 4. Targeting BMPs within the SHH-BMP signaling pathway is highly attractive, because the BMP molecules act extracellularly and, as such, can be easily reached by antibodies such as our recently developed highly selective low molecular weight llama VHHs. These VHHs specifically target BMP4 (C4C4) and BMP2/4 (C8C8) and are superior to conventional antibodies [35, 36]. In this study, we focused on evaluating the effects of inhibiting BMP4 and BMP2/4 in SMAD4 mutated cells in comparison to SMAD4 expressing cells. Hereto, we selected a unique recently established SMAD4(-) EAC xenograft-derived cell line, ISO76A, which we transfected with a wild type SMAD4 expression construct to obtain a ‘SMAD4(+) ISO76A’ counterpart.

To investigate whether cases with a low SMAD4 expression exhibit increased non-canonical signaling, we performed GSEA analysis and compared a subset of EAC cases with the highest SMAD4 expression to those with the lowest expression and found that one of the known BMP non-canonical signaling pathways, the p38 MAPK pathway, was significantly enriched. We confirmed involvement of this pathway by stimulating SMAD4(-) ISO76A cells with BMP2 and BMP4, which enhanced the expression of p38 and pERK. In contrast, stimulation of SMAD4(+) ISO76A cells by these BMPs only increased the normal canonical BMP signaling. These results are in agreement with a previous report using the colorectal cell lines SMAD4(+) HCT116 and SMAD4(-) HCT116 [7]. In contrast to other reports, BMP stimulation had no significant effect on the expression of p-NFkB. Also, BMP stimulation did not induce any changes in EMT markers. Thus, SMAD4 mutation and/or loss in EAC seems to be the switch that changes BMP signaling from tumor-suppressive canonical BMP signaling, to tumor-promoting non-canonical BMP signaling.

In search of the upstream signals which induce BMP2/4 expression, we found a significant correlation between SHH, BMP2 and BMP4 through the analysis of the TCGA database. Using IHC, we confirmed the presence of SHH in both stromal and cancer cells of EAC, while BMP2 and 4 were mostly expressed in the cytoplasm of the tumor cells. Upregulated SHH signaling is often observed in EAC [52–54], but also in its precursor lesion known as BE [4]. SHH is known to upregulate several types of BMPs including BMP4 [55] and can be produced by cancer-associated fibroblasts in the cancer microenvironment [12]. Under in vitro conditions, we found that cultured tumor cells, in the absence of a stromal compartment, failed to express BMPs and that this can be overcome under the influence of SHH. In addition, we not only demonstrated that SHH primarily induces BMP2 and BMP4 expression in ISO76A cells, but also that high SHH, BMP2 and 4 expression increases the chemoresistance of SMAD4(-) ISO76A cells. This is in line with a previous report [56], which demonstrated that BMP4 is highly expressed in cisplatin-resistant gastric cancer cells and that targeting BMP4 sensitizes these cells to cisplatin. Of importance is that in our cell viability assay we showed that the chemoresistance induced by SHH, or directly by BMP2 and 4 stimulation, could be rescued by inhibiting BMP4 and BMP2/4 using C4C4 and C8C8, respectively. These effects were not seen in SMAD4(+) ISO76A cells. Patients with EAC notoriously exhibit a varied response to chemotherapy and they are relatively chemo resistant. Therefore, targeted therapies which can overcome chemoresistance are of importance.

EAC is also a highly metastatic cancer, which accounts for the high recurrence and mortality rates of this disease. Cancer cell migration assays can be used to investigate a cancer cell’s capacity to infiltrate in neighbouring tissues, to enter lymphatic and blood vessels and to disseminate into the circulation [57]. We investigated whether the effects of the BMP inhibition would reduce the migration of ISO76A cells. Indeed, our migration assay indicated that BMP2/4 accelerated the migration of SMAD4(-) ISO76A cells, which could be effectively inhibited by our VHHs. These effects were absent in SMAD4(+) ISO76A cells.

The effects on migration and chemoresistance of the VHHs on the SMAD4 negative EAC cells, prompted us to further study the effects of the VHHs in vivo using SMAD4(-) ISO76A cells in a PDX model in NSG mice. This allowed us to investigate the anti-cancer effect of VHHs in an intact tumor micro-environment. IHC revealed that SHH, BMP2 and BMP4 were expressed in this PDX model. Delivery and effective targeting of the PDXs in this model was confirmed in real time through in vivo imaging using IRDye 800CW labeled VHHs and by ex vivo imaging of organs. We found that the labeled VHHs reached tumor sites within 30 minutes post tail vein injection and retention of VHHs in tumor sites was still observed 24 hours post injection. These results are in agreement with the physiological properties of the VHHs, which have a high target affinity and binding [35, 36]. Also, rapid secretion of VHHs via kidneys and liver was observed two hours post injection, which is thought to be related to the low molecular weight of the VHHs.

Finally, we studied the tumor response in terms of tumor size by systemic administration of the VHHs. We found that the VHHs used alone, or in combination with cisplatin, significantly inhibited tumor growth. The combination of the VHHs with cisplatin showed a further decrease in tumor growth, which suggests that there is a synergetic effect between the VHHs and cisplatin in inhibiting tumor growth. These results are in line with our cell viability assay indicating that the VHHs decreased chemo-resistance to cisplatin. It is well known that cisplatin as an anticancer drug has unavoidable side effects in patients and animals [58]. One of these side effects is mucosal damage, which can appear anywhere within the gastro-intestinal tract [58, 59]. In the present study, we investigated the organs of the animals only at the end of the study period. At this time point, we found that the number of goblet cells in the colon of the mice were severely reduced in those treated with cisplatin. This effect was not seen in the group treated with the VHHs. Even more interesting is that the combination of the VHHs with cisplatin did not result in this side effect. This could mean that the anti-BMP VHHs protect against the side effects of cisplatin. The mechanism behind this observation needs to be further clarified. Another future priority includes further assessment of the potential side effects of VHHs, both when administered alone and in combination with chemotherapeutic agents.

In summary, through data analysis, imaging and functional assays in in vitro and in vivo preclinical models, we demonstrated the anti-cancer effects and feasibility of selectively targeting BMP2/4 in SMAD4 negative EAC by novel anti BMP4 and BMP2/4 VHHs. To translate these findings to the clinic, future patient trials are required. We anticipate that specific targeting of BMP2/4 using the VHHs could form a basis for personalised treatment in SMAD4 deficient EAC, and perhaps in other SMAD4 mutated cancers. Based on the present data, we believe that targeting BMP4 and BMP2/4 can potentially improve clinical outcomes of the highly aggressive SMAD4 deficient subgroup of EAC.

## Conclusions

Overexpression of BMP2/4 triggers non-canonical BMP signaling and aggressive behaviour in SMAD4 negative EAC. Inhibition of BMP2/4 by VHHs decreases malignant behavior and improves survival. Therefore, targeting BMP2/4 with VHHs is promising as a novel and personalized treatment option for SMAD4 negative EAC.

## Supplementary information

Supplementary Fig. 1 (**A**) Luciferase analysis of BMP activity in ISO76A, Flo-1, OE19, OE33, SKGT4, OACM5 and OACP4 cells(p<0.01). Results are relative to the baseline level of the medium (PNG 30 kb)

High resolution image (TIF 5524 kb)

Supplementary Fig. 2 (**A**) pSMAD1/5/8, pERK1/2, p38, and E-cadherin levels in SMAD4(+) ISO76A cells stimulated by BMP2(100 ng/ml) or BMP4(100 ng/ml) for 0’, 5’, 10’, 15’, 20’, and 25’. (**Aii**) Quantification of outputs as observed in (**Ai**). GAPDH served as loading control (PNG 414 kb)

High resolution image (TIF 16830 kb)

Supplementary Fig. 3 (**A**) SMAD4(-) ISO76A cells were separately stimulated by BMP2(100 ng/ml) and BMP4(100 ng/ml), for 0’, 5’, 10’, 15’, 20’, and 25’. (**Aii**) Quantification of outputs as observed in (**Ai**). GAPDH served as loading control (PNG 514 kb)

High resolution image (TIF 16362 kb)

Supplementary Fig. 4 (**Ai**) Tumor growth curves of C4C4 alone and in combination with cisplatin in mice 28 days. (**Aii**) Tumor growth curves of C8C8 alone and in combination with cisplatin in mice 28 days (PNG 121 kb)

High resolution image (TIF 17360 kb)

Supplementary Fig. 5 Ex vivo evaluation of the PDTX’ by H&E and IHC analysis for BMP2, BMP4, SMAD4, and SHH of the tumor, lung, and colon from the PDTX’. H&E staining(first panel) and IHC for BMP2, second panel; BMP4, third panel; SMAD4, fourth panel, SHH, fifth panel of the dissected tissues. Tissues were magnified by a factor of ten(**i, ii, iii, iv, v**), twenty(**vi, vii, viii, ix, x**) and forty( **xi, xii, xiii, xiv, xv**). Representative result of (**A**) tumor, (**B**) lung, and (**C**) colon (PNG 7616 kb)

High resolution image (TIF 53060 kb)

Supplementary File 1 (XLSX 113 kb)

Supplementary File 2 (TXT 21 kb)

Supplementary File 3 (XLSX 46 kb)

Supplementary Table 1 (XLSX 11 kb)

Supplementary Table 2 (XLSX 10 kb)

ESM 1 (7Z 6911 kb)

## Data Availability

The data used in this study can be obtained by request to Shulin Li (s.li@amsterdamumc.nl ).
