# Supplementary figures and images for: Selective targeting BMP2 and 4 in SMAD4 negative esophageal adenocarcinoma inhibits tumor growth and aggressiveness in preclinical models
Source: Cell Oncol (Dordr). 2022 Jul 28;45(4):639–58. doi: 10.1007/s13402-022-00689-2 (PMC9333053; doi:10.1007/s13402-022-00689-2)

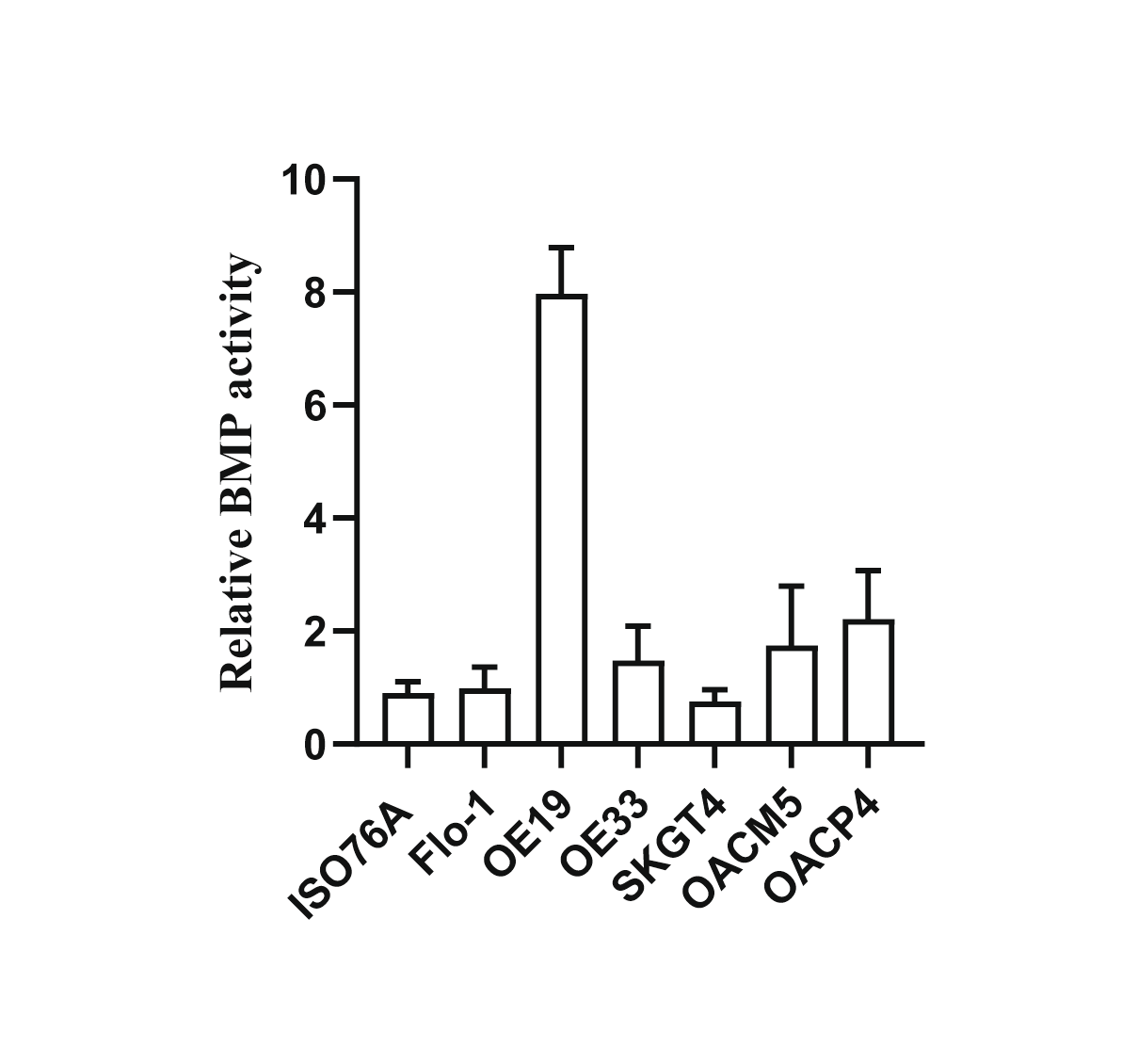

Supplement: Supplementary file 1 — (A) Luciferase analysis of BMP activity in ISO76A, Flo-1, OE19, OE33, SKGT4, OACM5 and OACP4 cells(p<0.01). Results are relative to the baseline level of the medium (PNG 30 kb) [file 13402_2022_689_Fig8_ESM.png]

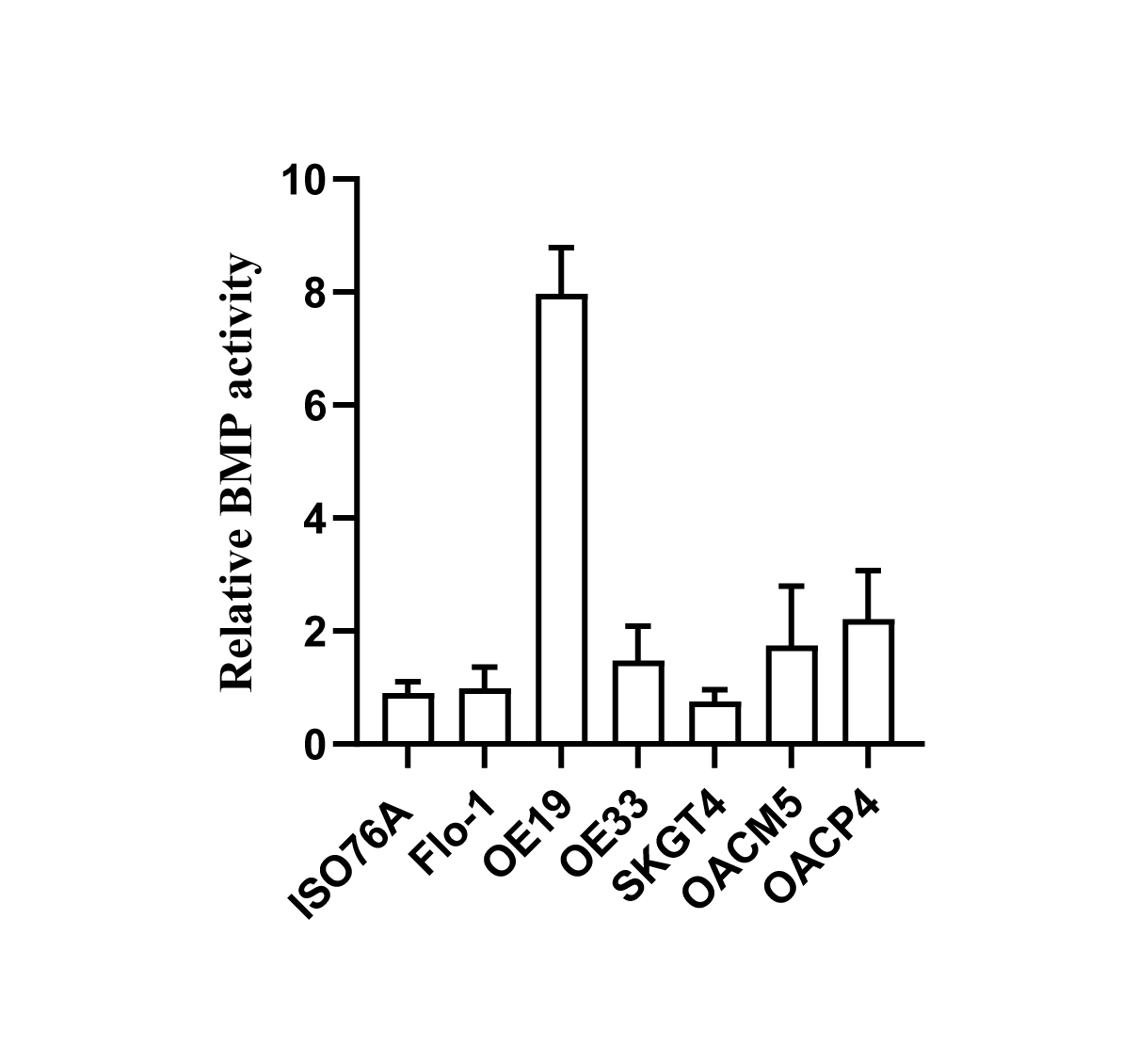

Supplement: Supplementary file 2 — High resolution image (TIF 5524 kb) [file 13402_2022_689_MOESM1_ESM.tif]

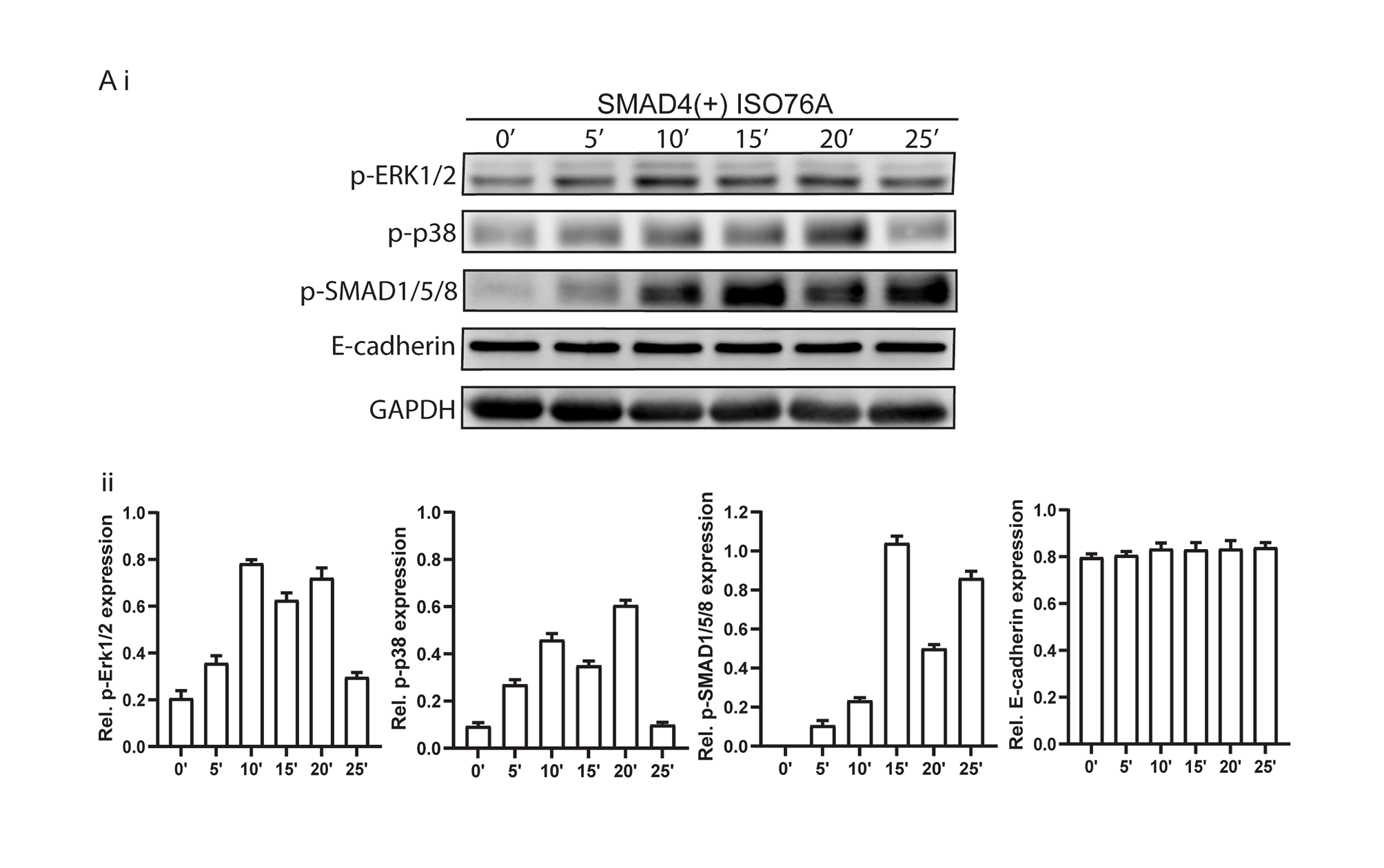

Supplement: Supplementary file 3 — (A) pSMAD1/5/8, pERK1/2, p38, and E-cadherin levels in SMAD4(+) ISO76A cells stimulated by BMP2(100 ng/ml) or BMP4(100 ng/ml) for 0’, 5’, 10’, 15’, 20’, and 25’. (Aii) Quantification of outputs as observed in (Ai). GAPDH served as loading control (PNG 414 kb) [file 13402_2022_689_Fig9_ESM.png]

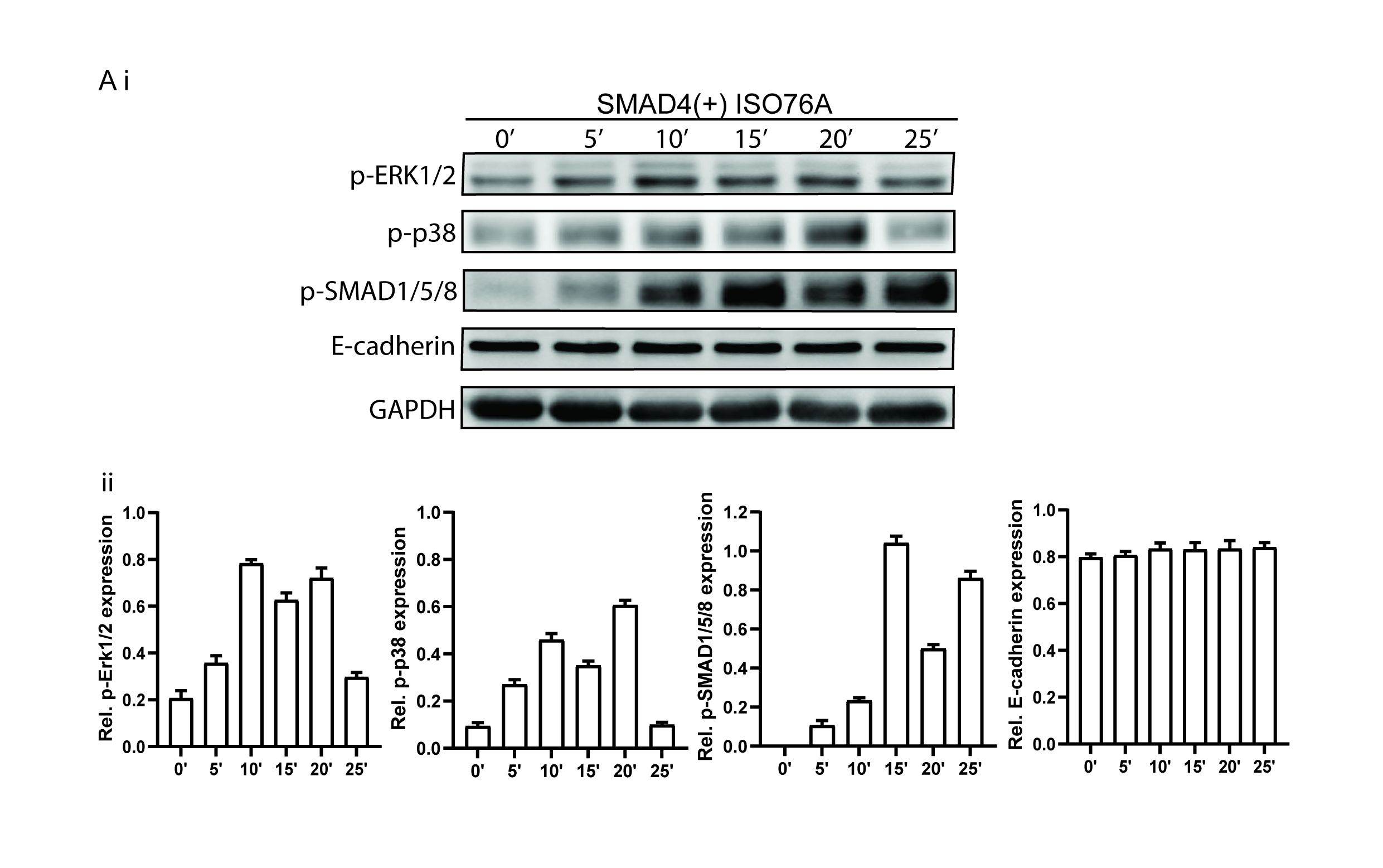

Supplement: Supplementary file 4 — High resolution image (TIF 16830 kb) [file 13402_2022_689_MOESM2_ESM.tif]

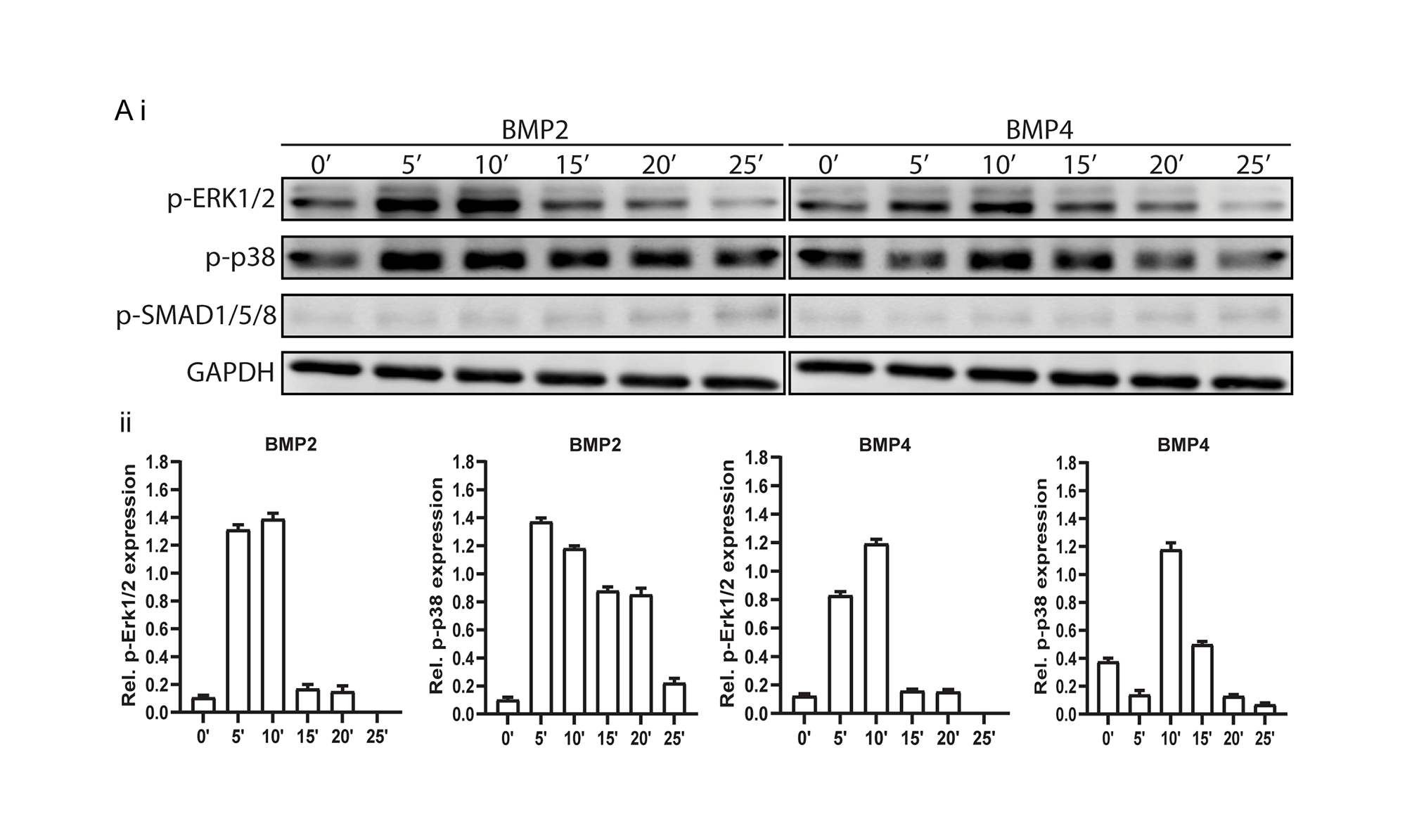

Supplement: Supplementary file 5 — (A) SMAD4(-) ISO76A cells were separately stimulated by BMP2(100 ng/ml) and BMP4(100 ng/ml), for 0’, 5’, 10’, 15’, 20’, and 25’. (Aii) Quantification of outputs as observed in (Ai). GAPDH served as loading control (PNG 514 kb) [file 13402_2022_689_Fig10_ESM.png]

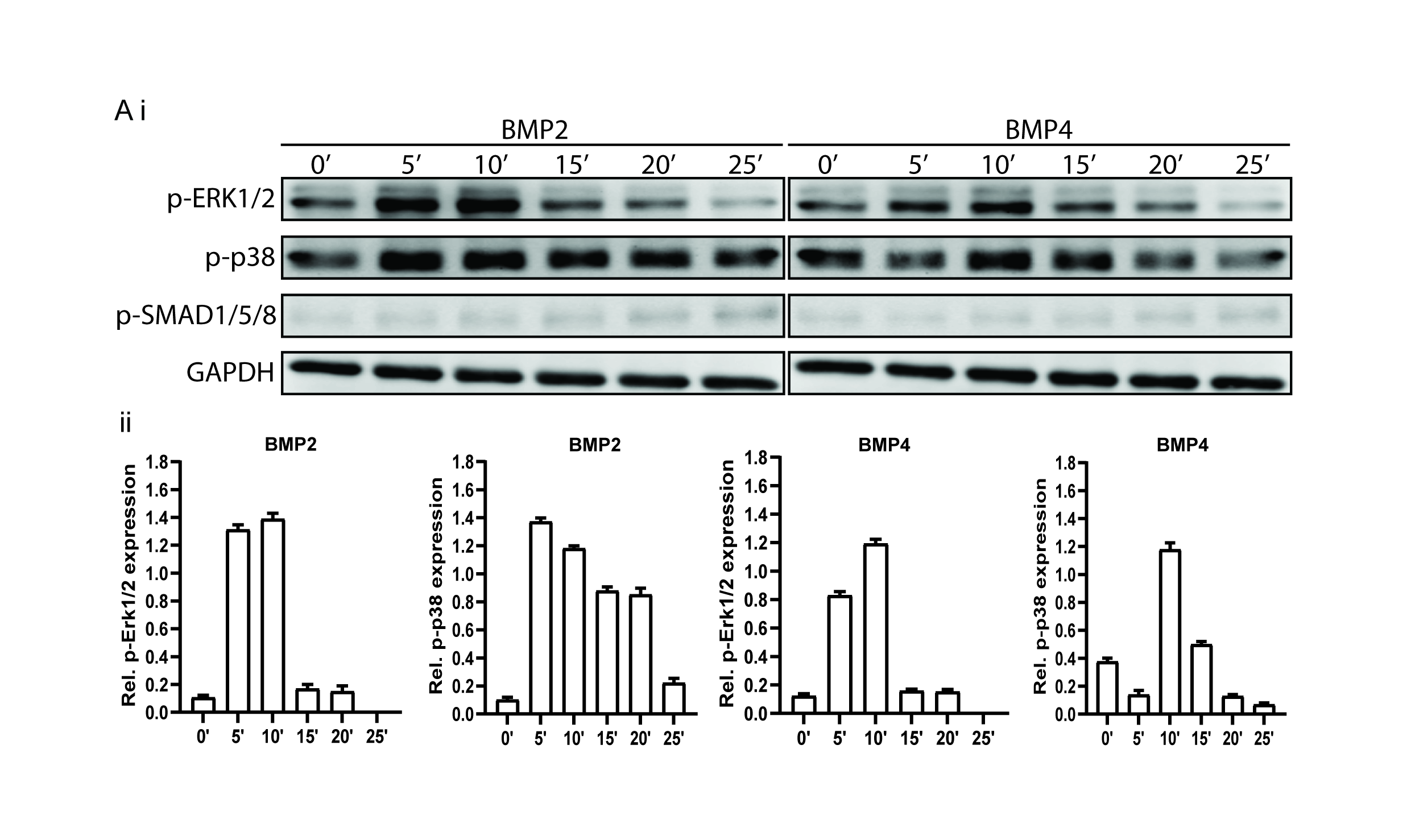

Supplement: Supplementary file 6 — High resolution image (TIF 16362 kb) [file 13402_2022_689_MOESM3_ESM.tif]

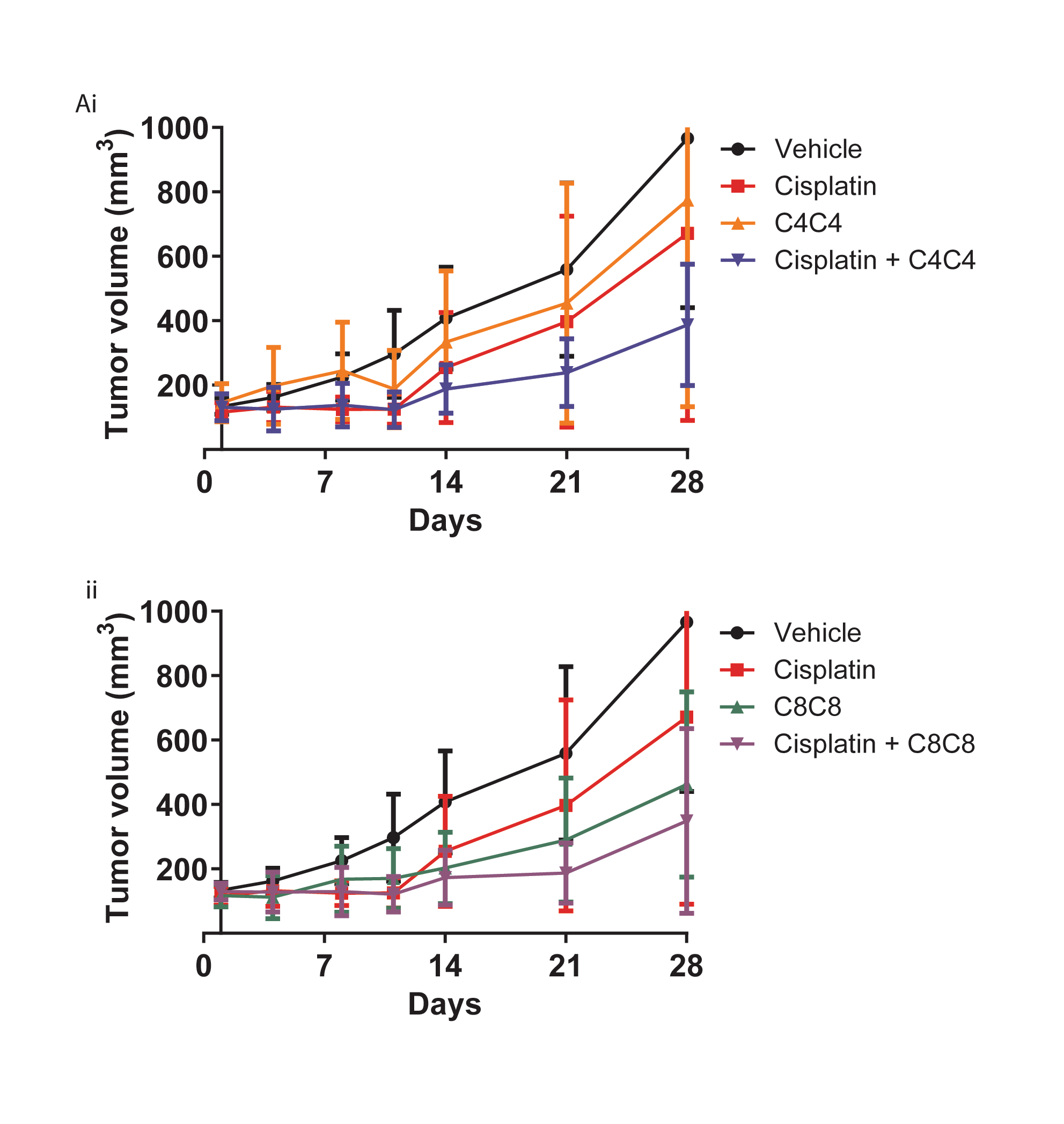

Supplement: Supplementary file 7 — (Ai) Tumor growth curves of C4C4 alone and in combination with cisplatin in mice 28 days. (Aii) Tumor growth curves of C8C8 alone and in combination with cisplatin in mice 28 days (PNG 121 kb) [file 13402_2022_689_Fig11_ESM.png]

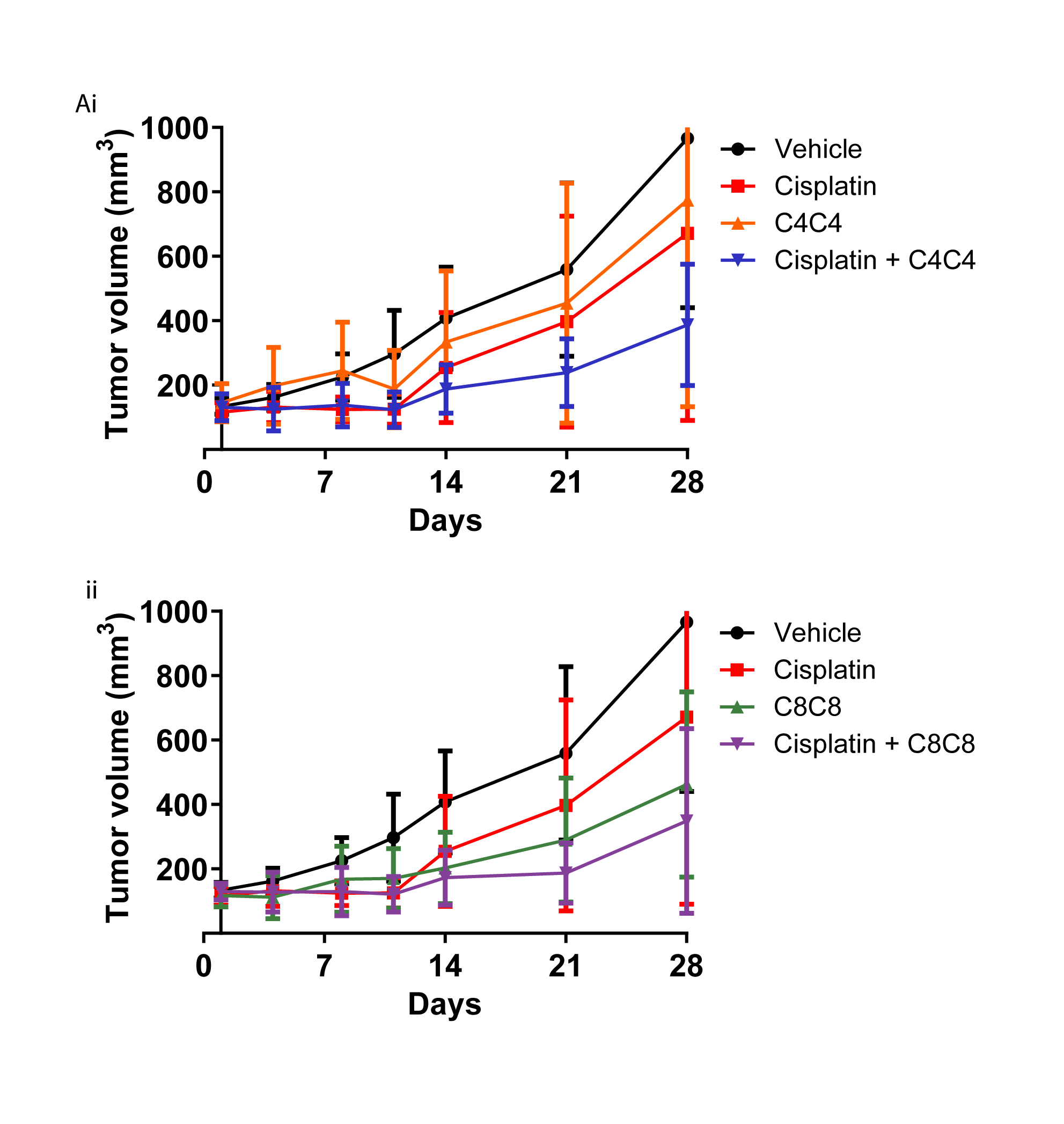

Supplement: Supplementary file 8 — High resolution image (TIF 17360 kb) [file 13402_2022_689_MOESM4_ESM.tif]

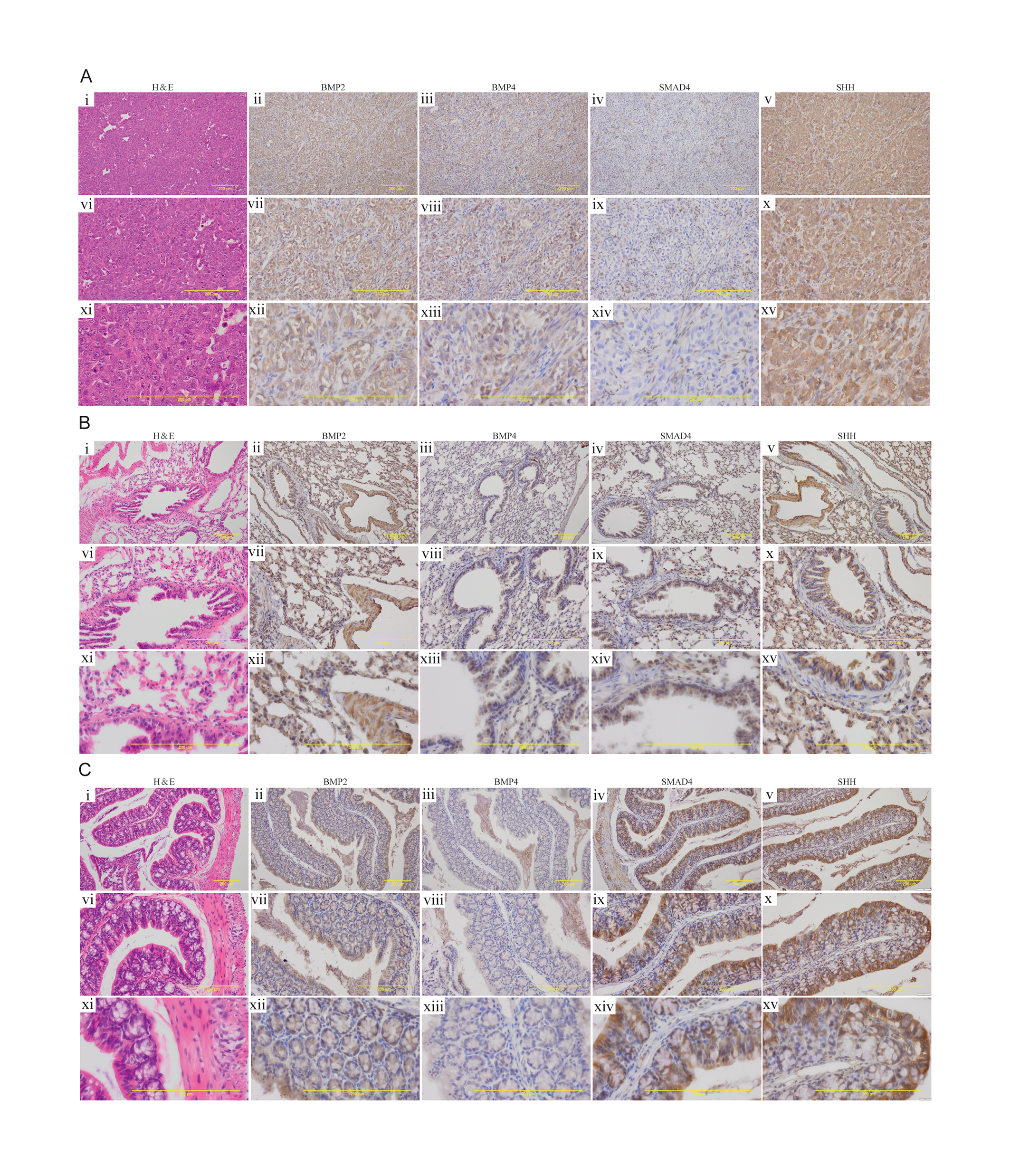

Supplement: Supplementary file 9 — Ex vivo evaluation of the PDTX’ by H&E and IHC analysis for BMP2, BMP4, SMAD4, and SHH of the tumor, lung, and colon from the PDTX’. H&E staining(first panel) and IHC for BMP2, second panel; BMP4, third panel; SMAD4, fourth panel, SHH, fifth panel of the dissected tissues. Tissues were magnified by a factor of ten(i, ii, iii, iv, v), twenty(vi, vii, viii, ix, x) and forty( xi, xii, xiii, xiv, xv). Representative result of (A) tumor, (B) lung, and (C) colon (PNG 7616 kb) [file 13402_2022_689_Fig12_ESM.png]

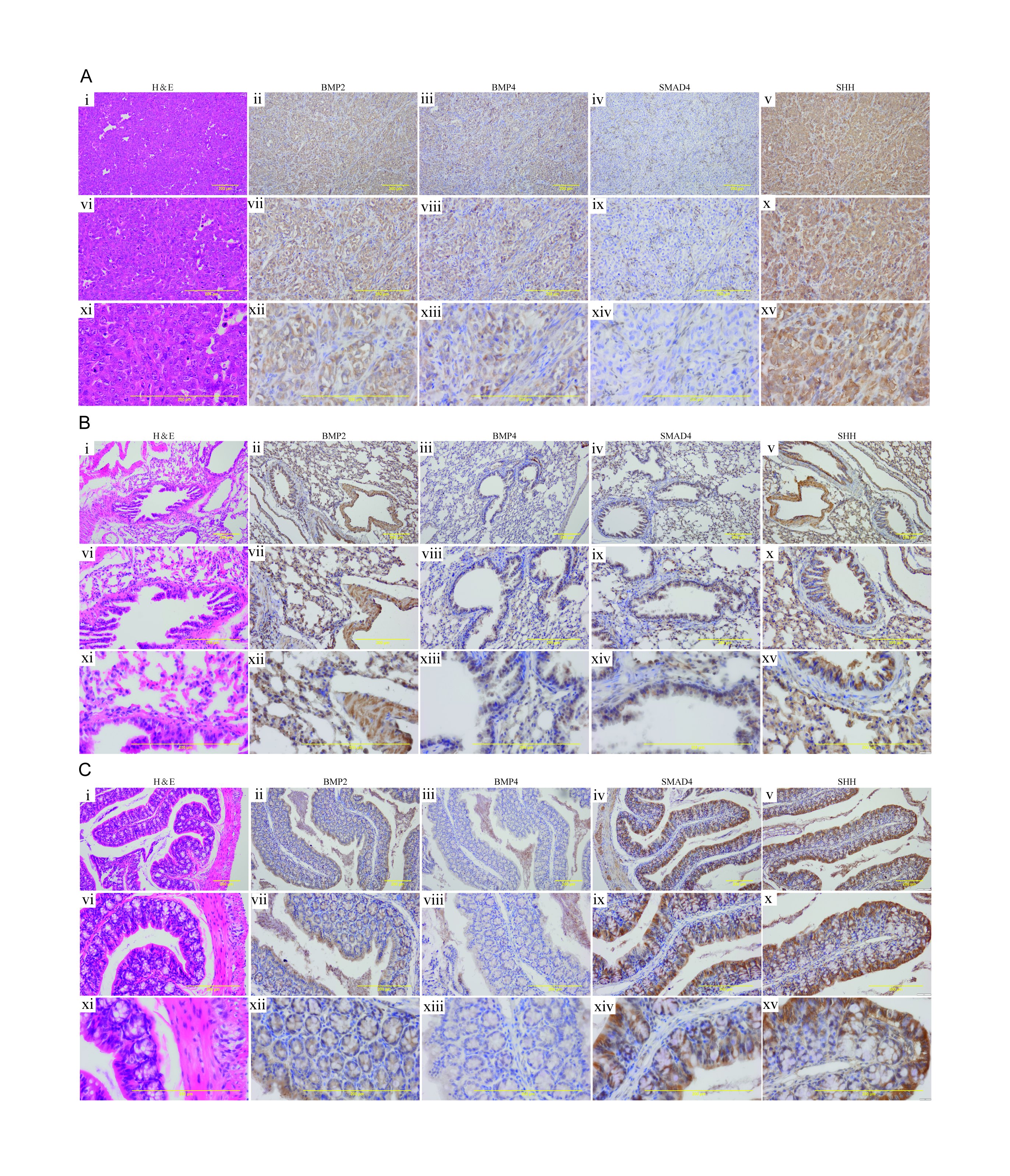

Supplement: Supplementary file 10 — High resolution image (TIF 53060 kb) [file 13402_2022_689_MOESM5_ESM.tif]
